# COVID-19-related changes in eating disorder pathology, emotional and binge eating and need for care: a systematic review with frequentist and Bayesian meta-analyses

**DOI:** 10.1007/s40519-023-01547-2

**Published:** 2023-02-20

**Authors:** Âmine Güzel, Naz Lâl Mutlu, Marc Molendijk

**Affiliations:** 1 Clinical Psychology Department, Leiden University, Leiden, The Netherlands; 2 Leiden Institute of Brain and Cognition, Leiden University Medical Center, Leiden, The Netherlands

**Keywords:** Eating disorders, Anorexia nervosa, Bulimia nervosa, Binge eating, Emotional eating, COVID-19, Corona, Lockdown

## Abstract

**Purpose:**

The COVID-19 pandemic has been a leading cause of stress and feelings of loss of control, both of which have been related to eating disorder (ED) pathology onset and deterioration. We aim to estimate the magnitude of changes in the prevalence rates of, and indicators for, ED psychopathology in the face of the COVID-19 pandemic.

**Method:**

Pre-registered systematic review with frequentist and Bayesian meta-analyses. Searches for eligible studies were performed in PubMed, Web of Science and pre-print servers until January 15 2023.

**Results:**

Our searches yielded 46 eligible studies reporting on a total of 4,688,559 subjects. These data provide strong evidence indicating increased rates of diagnosed and self-reported ED’s and a concordant increased need for care in the face of the pandemic. ED symptom severity scores in patients were not elevated during the pandemic, except for those related to anorexia nervosa. On average, people in the general population report relatively high levels of emotional and binge eating during the pandemic, although the evidential strength for these associations is only anecdotal to moderate. Moderators of between-study heterogeneity were not detected.

**Conclusions:**

Altogether, our results suggest that the COVID-19 pandemic is associated with a wide spread negative effect on ED pathology in patient samples and the general population. The development of online prevention and intervention programs for EDs during stressful times like a pandemic is encouraged. A limitation is that the results reported here may be prone to biases, amongst others, self-report bias.

Level of evidence: Level I, systematic review and meta-analysis.

Preregistration: Prospero [https://www.crd.york.ac.uk/prospero] ID: CRD42022316105.

**Supplementary Information:**

The online version contains supplementary material available at 10.1007/s40519-023-01547-2.

## Introduction

In December 2019, the SARS‑CoV‑2 abruptly spread worldwide, causing the coronavirus disease (COVID-19) pandemic [1, 2]. This pandemic has proven to be a huge source of stress and caused a profound disruption in millions of people's life for multiple reasons (World Health Organization, 2022 [3]). A worsening in mental health symptoms and increased rates of psychopathology has been observed following the start of the pandemic in children, adolescents, and adults [4–6].

The onset of eating disorders (ED) and related behaviors such as binge eating (BE) and emotional eating (EE) in general population and at-risk samples has been related to stress exposure and adversity [7–9]. Theories even suggest that pathological eating behaviors can serve as a coping response to deal with stress and adverse emotions [10, 11]. Given the stress generating nature of the COVID-19 pandemic and its profound impact on emotional wellbeing, alternations in the prevalence and severity of EDs and related behaviors are to be expected, together with a concordant increase in the need for ED-related care. Indeed, several studies show such trends. At the diagnostic level, studies exist that report increases in the rates of anorexia nervosa (AN) and bulimia nervosa (BN) [12, 13]. A worsening of symptoms of individuals with ED’s and higher rates of BE and EE has been reported as well in people with AN and BN [14, 15]. In line with this are findings of an increased demand for care related to ED, such as an increased hospitalization rate due to AN [16–18].

We know of six systematic reviews on the topic [12, 19–23]. Five of them [12, 20–23] sketch the picture that ED pathology worsened in the general population and in AN and BN patient populations and that related care demand increased with the advent of the pandemic. One of the reviews, however, concludes that the evidence for Covid-19’s impact on ED psychopathology in the general population and in patients is limited due to methodological weaknesses of the available data [19]. To our knowledge, four quantitative meta-analyses were performed on this topic [24–27]. Sidelli et al*.* [24] found that about 50% of people with an ED (AN, BN, or BED) reported a worsening of symptoms in relation to the pandemic. Similar findings, yet based on a larger database, were reported by Haghshomar et al*.* [25]. Khraisat et al*.* [26] also reports an overall increase in ED symptoms, and beyond that highlights a significant increase in comorbid anxiety and depression symptoms in people with an ED diagnosis. Based on 11 studies, Gao et al*.* [27] report an overall symptom deterioration. Associated factors, amongst which anxiety and social isolation, are also reported on by these authors.

Many relevant studies have been published since the publication of the excellent meta-analyses, specified above and hence an update seems relevant. In an updated meta-analysis, we see fit for the assessment of effects of timing of the pandemic (i.e.*,* whether observations are similar in the early phase of the pandemic, relative to later phases). Assessment on pandemic related changes in prevalence rates and symptom severity of specifically AN, BN and BED and of changes in BE and EE also seem relevant add-ons. Finally, we want to sketch a broad picture of the pandemic effects on ED patients and hence we will also meta-analyze its effects on comorbid mental health syndromes and symptoms (e.g.*,* suicidality) and on the use of care by ED patients (e.g.*,* emergency room visits for ED symptoms). Finally, we aim to summarize the existing data on changes in EE and BE in the general population in the face of the pandemic. We find this relevant in order to shed some further light on the continuum of eating behaviors and EDs [28].

Overall, we hypothesize a wide spread negative effect of the pandemic on ED-related pathology, related behaviors, need for care, and psychiatric comorbidity. We will present findings from both classical (i.e.*,* frequentist) and Bayesian meta-analysis because these approaches complement each other in achieving our goals. The classical meta-analyses will provide the option for significance testing of the null hypothesis (i.e.*,* the hypothesis that the pandemic had no effect on outcome). The Bayesian approach will allow us to discern whether there is “evidence of absence of an effect” versus “absence of evidence”, that is whether the data ara inconclusive or whether there is evidence suggesting no effect of the pandemic on the outcomes of interest [29]. The knowledge that we will generate may contribute to theory on the origins of pathological eating behaviors and could inform policy regarding the development of prevention and intervention programs.

## Method

Our method and approach were carried out in compliance with the Meta-analysis Of Observational Studies in Epidemiology (MOOSE) group [30] and PRISMA guidelines [31]. The PRISMA checklist for this study is presented as Supplementary Material. This meta-analysis has been pre-registered In the International Prospective Register of Systematic Reviews (PROSPERO, ID CRD42022316105).

### Search and selection strategy

PubMed and Web of Science were systematically searched for eligible studies. Systematic searches were supplemented with a non-systematic search in Google Scholar. A grey literature search on the preprint servers Psyrxiv.org and Biorxiv.org was performed. Data bases were searched from their inception. Official searches started March 19 2022 and final search date was January 15, 2023. The following search terms were used; ((Covid* OR Corona OR SarS* OR Lock down) AND ("emotional eating" OR binge* OR eating OR Bulim* OR Anorex*)). The researchers independently conducted the literature search. After screening and first selection, full text versions of the articles were evaluated to come to a decision on final inclusion. We applied the inclusion and exclusion criteria (see below) in making this decision. Cases of disagreement were resolved through discussion and consensus.

### Inclusion and exclusion criteria

Articles were included when they (1) were case–control studies, retrospective cohort studies, and prospective cohort studies, (2) were written in English, Dutch, Spanish, Turkish, German, or French, (3) reported on changes in ED pathology and related behaviors in humans of all ages (see below under data extraction) pre versus during the COVID-19 pandemic or at several time points within the pandemic. Articles were excluded when they were not based on original data (e.g.*,* reviews). When data related to our study were missing where it was expected that such data were gathered in a study, the data were requested by contacting the corresponding author of that particular study. In case data could not be obtained after this request, the study was excluded. This occurred in four instances (see Table S1).

### Data extraction

Study selection and data extraction were performed independently by all authors. We extracted data on the total number of participants, year and month of data gathering, demographic information (mean age in years, gender distribution, the country in which the study was performed), clinical information (method of diagnostic assessment, type of disorder, and the presence of symptoms at baseline and follow-up), and outcome data (raw numbers or effect-size estimates and their corresponding 95% confidence interval. For patient samples these include the following outcome variables: (I) prevalence rates of AN, BN, and BED as assessed pre- and during the pandemic, (II) cross-sectional data on self-reported changes in ED symptomatology (e.g.*,* pre-clinical symptoms emerged/disappeared in the face of the pandemic), (III) questionnaire data on changes in the severity of ED symptoms as assessed pre- and during the pandemic, (IV) comorbid depression, anxiety and suicidality assessed by different means pre- and during the pandemic, (V) need for care and access to care, including age at admission and duration of admission (e.g.*,* number of hospital days) and clinical impairment pre- versus during the pandemic. For non-patient samples we extracted (VI) questionnaire data on changes in the frequency and intensity of EE and BE assessed pre- versus during the pandemic.

### Assessment of methodological quality

Retained articles were assessed on methodological quality. This was done independently by all authors using the method proposed by Lievense et al*.* [32]. The items that compose this unidimensional scale are presented in Table S2. Methodological quality of the articles did not serve as in or exclusion criterion. Rather, continuous methodological quality scores were related to outcomes in moderator analyses.

### Statistical analysis

Meta-analyses were conducted in Stata version 17 [33] and JASP. Summary tables on the characteristics of included articles were created. Separate random-effects meta-analyses [34] were conducted on the 6 outcomes reported under data extraction (I–VI). In case a meta-analysis was conducted on different disorder types, e.g.*,* AN and BED, sensitivity analyses were performed. When there was sufficient data, we also investigated differences in outcome during the pandemic, defined as first year versus second year. Outcomes were reported as weighted and pooled odds ratios (OR) in case of categorical outcome variables, Cohen’s *d* for continuous outcome variables, and weighted proportions for proportional data. All outcomes were reported with corresponding 95% confidence intervals (CI). Between-study heterogeneity was expressed as *I*^2^ and tested for statistical significance using the Q statistic [35]. Statistical significance was set at *P* < 0.05. We repeated all analyses using a Bayesian approach. From these analyses we reported the results from the random-effects model and the averaged Bayes Factor (BF_10_) for priors of *d* = 0 or Odds Ratio (OR) = 1 and with scale parameter 0.5 as main outcome. For one-sided hypothesis testing, we truncated values below 0 for Cohen’s d and below 1 for OR’s. BF’s were interpreted according to the suggestions provided by Morey and Rouder [36]. Moderator analyses were performed in case of statistically significant between-study heterogeneity on outcomes that were based on > 5 independent studies. The tested moderators were: (I) gender, operationalized as percentage women in a given sample, (II) age operationalized as adolescent samples *versus* adult samples, and (III) methodological quality. Gender and age were assessed as effect-moderators because these variables are strongly related to variance in ED onset and course [37, 38]. Methodological quality was related to outcome since variance in this variable can be related to effect size estimates, maybe notably so in COVID-19 related studies [39]. Estimates of publication bias were generated by means of Egger's regression test for the assessment of funnel plot asymmetry [35].

## Results

**Fig. 1:**
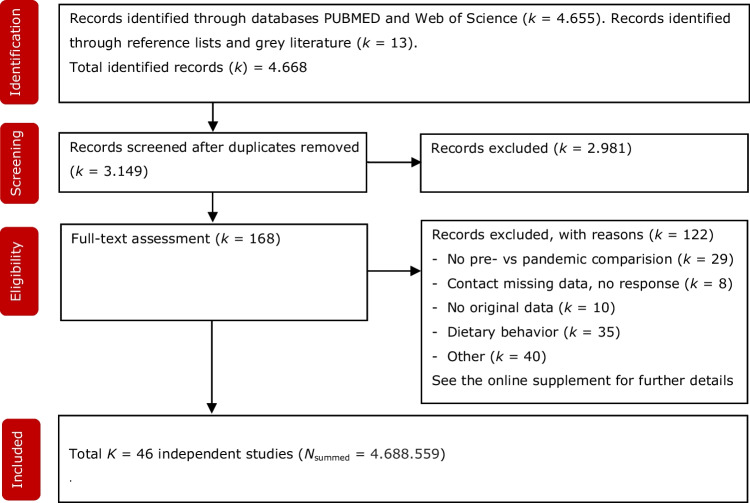
Flowchart on identification, screening and inclusion of eligible publications

Overall, 2446 records were identified after the removal of duplicates. After screening these records, 149 records were assessed in full text for eligibility. Interrater agreement on first selection was high (Cohens Kappa = 0.74; SE = 0.05). Based on a comment by a reviewer we reran the search with the term OSFED included. This did not lead to additional hits. Figure 1 shows the flowchart and outlines the information on the identification, screening, and inclusion of record. A total of 46 independent studies were included in the review, reporting on a total of 4,688,559 subjects.

Average ages of the included samples ranged from 7 to 65 years (average age per sample = 23 years). Women were overrepresented in 42 of the 46 included studies (average percentage of women = 80%). Data were gathered in 16 different countries. Western countries yielded most input samples (i.e., 7 from the USA, 6 from Germany, and 6 from Italy). There was no eligible data from Asia (except from Turkey), Africa, or South America. Key characteristics of the included studies are presented in Table 1. Reasons for exclusion in the second round of article selection are provided in Table S1. Methods with which symptom severity was assessed are presented in Table S2.

**Table 1:** Characteristics of included samples

Study	ED	Age^1^	% Female	Country	*N*	Meta-analysis
Albert et al. 2021 [16]	BE	48	68	Italy	56	II, VI
Allgaier et al. 2022 [40]	MIX	14	N/K	Germany	47	V
Baenas et al. 2021 [14]	AN, BN	28	70	Mix	829	II, IV
Branley-Bell and Talbot 2020 [41]	AN, BN	16–65	94	UK	129	III
Bianchi et al., 2022 [42]	BE	24	72	Italy	1925	VI
Breiner et al. 2021 [43]	BE	28	91	USA	159	VI
Carison et al. 2022 [44]	ED	7–18	72	Australia	30,170	V
Castellini et al. 2020 [45]	AN, BN	31	100	Italy	171	II, VI
Christensen et al. 2020 [46]	AN, BN	21	75	US	579	I
Colleluori et al. 2021 [47]	AN, BN	N/K	N/K	Italy	453	III
Datta et al. 2022 [48]	AN	15	96	Australia	50	II, IV
Elmacioglu et al., 2020 [49]	EE	33	80	Turkey	1036	VI
Eray and Sahin 2021 [50]	MIX	14	57	Turkey	272	I
Favreau et al., 2021 [51]	AN, BN	NK	70	Germany	113	III
Feldman et al., 2022 [52]	MIX	14	71	USA	71	IV
Freitas et al., 2021 [53]	None	21	100	Brasil	71	VI
Giel et al. 2021 [54]	MIX	42	81	Germany	34	II, IV
Goldberg et al. 2022 [55]	AN, BN	15	86	Isreal	275	II, V
Graell et al. 2020 [56]	AN	14	100	Spain	1818	IV, V
Hansen et al. 2021 [57]	MIX	15 and 28	98	New Zealand	121	V
Haripersad et al. 2021 [58]	AN	N/K	N/K	Australia	134	I
Jones et al. 2020 [18]	MIX	N/K	N/K	Australia	243	V
Kersten et al. 2023 [59]	MIX	0 to 18	N/K	The Netherlands	47,621	IV
Kim et al. 2021 [60]	MIX	19	29	USA	8613	I
Klump et al. 2022 [61]	BE	22	100	USA	402	VI
Machado et al. 2020 [62]	MIX	28	95	Portugal	43	I
Marino et al. 2021 [63]	AN	N/K	N/K	Italy	1050	I
Martinez de Quel et al. 2021 [64]	MIX	35	37	Spain	161	I, II
Matthews et al. 2021 [65]	AN	15	83	USA	163	V
Miskovic‐Wheatley et al. 2022 [15]	AN, BN	25	92	Australia	1723	II, VI
Özcan and Yeşilkaya 2021 [66]	AN, BN, EE	32	73	Turkey	578	VI
Richardson et al. 2020 [67]	MIX	N/K	79	Canada	609	II, IV, VI
Schelhorn et al. 2021 [68]	MIX	34	71	Germany	3387	I
Schlegl et al. 2020 (a) [69]	AN, BED	24	100	Germany	159	III
Schlegl et al. 2020 (b) [70]	BN, BED	22	100	Germany	55	III
Spettigue et al. 2021 [17]	AN, BN, BED	15	74	Canada	91	II, IV, V, VI
Spina et al. 2022 [71]	AN, BN, BED	14	86	Italy	211	V
Springall et al. 2021 [72]	AN, BN	15	90	Australia	457	II, IV, V
Surén et al. 2022 [73]	AN, BN, BED	11	100	Norway	702,035	V
Takakura et al. 2022 [74]	MIX	20	100	Japan	148	II, V
Taquet et al. 2022 [75]	AN	15	44	USA	18,313	I, IV
Tazeoglu et al., 2021 [76]	EE	21	52	Turkey	386	VI
Termorshuizen et al. 2020 [77]	AN, BN, BED	31	98	MIX	1021	III
Toulany et al. 2022 [78]	MIX	10	49	Canada	3,862,051	V
Trott et al. 2021 [79]	MIX	36	84	UK	319	I, II
Vuilier et al. 2021 [80]	AN, BN, BED	30	64	UK	207	III

(I) changes in prevalence rates of AN, BN, and BED as assessed, (II) questionnaire data on changes in the severity of ED symptoms, (III) cross-sectional data on self-reported changes in ED symptomatology (e.g.*,* pre-clinical symptoms), (IV) changes in comorbid mental health depression, anxiety and suicidality, (V) changes in need for care and access to care, age at admission, and length of stay, and (VI) questionnaire data on changes in EE and BE

*AN* anorexia nervosa, *BE* binge eating, *BED* binge eating disorders, *BN* bulimia nervosa, *EE* emotional eating, *MIX* mixed category of disorders, *N/K* not known

^1^We report average age for included samples. In case these were not available, we report the reported range

### Meta-analyses on patient data

Random effects meta-analyses found an overall increase in prevalence rates of the ED’s during the pandemic, relative to those observed prior to the pandemic (Table 2). Bayesian meta-analysis confirmed this effect and showed very strong evidence favoring the alternative hypothesis over the null hypothesis of no effect (see Table S4, for Bayes Factors for the H_0_ and H_1_ and posterior probabilities). Given that virtually all samples were composed out of multiple types of EDs, we could not test whether the observed effect was due to a particular ED. Self-reported symptom prevalence rates were self-reported to be higher during the pandemic (see Table 2 for prevalence rates self-reported increases and decreases in AN and BN symptoms). In fact, studies that directly assessed within-subject in- and decreases showed that the odds of self-reporting an increase in symptom prevalence relative to a decrease are high for AN (OR = 9.58, 95% CI = 1.09 to 80.45, *k* = 5, *N* = 1394) and BN (OR = 9.21, 95% CI = 1.41 to 60.64, *k* = 6, *N* = 1364). These findings also were confirmed in Bayesian meta-analyses with Bayes Factors favoring H_1_ of 2504 and 2263, respectively.

**Table 2:** Mental health disorder and COVID-19 course variables from frequentist meta-analysis

ED patient data					
Prevalence rates ED	OR (95% CI)	*k*	*N*	*I*^*2*^	Egger’s test
AN / MIX ^1^	1.30 (1.05 to 1.54) ***	10	33,032	80.7 ***	0.77

ED symptom prevalence	Proportion (95% CI)	*k*	*N*	*I*^*2*^	Egger’s test
AN increase symptom prevalence	0.59 (0.39 to 0.80)	7	1483	98.7 ***	− 0.87
BN increase symptom prevalence	0.58 (0.42 to 0.74)	7	1376	91.9 ***	− 1.30
AN decrease symptom prevalence	0.23 (0.09 to 0.37)	5	1394	98.1 ***	0.66
BN decrease symptom prevalence	0.24 (0.13 to 0.38)	6	1346	97.0 ***	0.34

ED symptom severity	Cohen’s *d* (95% CI)	*k*	*N*	*I*^*2*^	Egger’s test
ED severity score	0.11 (− 0.06 to 0.28)	11	1875	86.8 ***	0.56
AN severity	0.29 (0.12 to 0.46) **	7	3095	50.7 ***	1.43
BN severity	0.24 (0.03 to 0.45) ***	7	2873	80.5 ***	1.73
BED severity	0.27 (0.02 to 0.43) *	4	2243	49.4	1.38

Comorbid psychiatric symptoms	Cohen’s *d* (95% CI)	*k*	*N*	*I*^*2*^	Egger’s test
*Depression*	0.21 (0.07 to 0.35) **	10	2012	67.6 **	0.23
*Anxiety*	0.23 (− 0.01 to 0.47)	9	1970	0.10	0.17
*Suicidality*	0.10 (− 0.42 to 0.61)	3	8722	41.4	0.16

Need for care and impairment	OR (95% CI)	*k*	*N*	*I*^*2*^	Egger’s test
Mix care category ^2^	1.74 (1.48 to 2.00) ***	13	5,260,823	83.3***	3.64 ** ^3^
Hospitalization	1.98 (1.37 to 1.48) ***	4	4,862,668	56.8	1.24

	Cohen’s *d* (95% CI)	*k*	*N*	*I*^*2*^	Egger’s test
Length of stay	0.19 (− 0.47 to 0.85)	5	1099	95.4***	− 0.42
Age at admission	0.31 (− 0.04 to 0.61)	5	837	0.00	0.56

General population data					
ED-related behaviors^1^	Cohen’s *d* (95% CI)	*k*	*N*	*I*^2^	Egger’s test
Binge eating	0.14 (0.02 to 0.22) *** ^2^	9	4882	0.0	2.48* ^4^
Emotional eating	1.05 (− 0.03 to 2.75)	6	1091	99.0***	− 0.61

*AN* anorexia nervosa, *BED* binge eating disorder, *BN* bulimia nervosa, *ED* eating disorder, *I*^*2*^ amount of between-study heterogeneity, *k* number of effect-size estimates, *N* number of participants, *OR* odds ratio

*P < 0.05, **P < 0.01, ***P < 0.001

^1^All studies, except for 2, reported prevalence rates for mixed ED categories. Hence no analyses by ED subtypes were performed

^2^Most often a mix of emergency visits, planned need for expert care, and hospitalization

^3^Trim-and-fill analysis yielded a somewhat smaller, yet significant, effect size estimate (OR = 1.65, 95% CI = 1.40 to 1.89)

^4^Trim-and-fill analysis yielded a somewhat smaller, yet significant, effect size estimate (*d* = 0.12, 95% CI = 0.60 to 0.18)

AN, BN, and BED symptom severity were reported to be higher during the pandemic, but general severity was not (Table 2). Bayesian analyses provided anecdotal evidence favoring the null hypothesis of associations between the pandemic on ED symptom severity (see Table S4). Depression comorbidity increased significantly during the pandemic. Such effects were not observed for anxiety comorbidity and suicidality (see Table 2). In fact, for the latter outcome, results from Bayesian meta-analysis provided evidence for the H_0_ (Table S4).

Classical and Bayesian meta-analyses converged in their results on pandemic related changes in need for care and provide strong evidence that this increased during the pandemic. There were no significant associations between the pandemic and the average age at which patients sought care and duration of care. Bayesian meta-analyses on these two associations proved that the data are inconclusive, so without evidence for both the null and the alternative hypotheses (Table 2 and Table S4).

### Meta-analyses in non-patient samples

Our data show that both BE and EE increased in the general population samples, but only the change in BE reached statistical significance (Table 2). Bayesian meta-analyses show that the evidential strength for the existence of these associations is anecdotal to moderate (Table S4).

### Between-study heterogeneity, publication bias and effect-moderators

Between-study heterogeneity was observed in basically each meta-analysis that we performed. There were no moderators that consistently explained variance in this heterogeneity (Table S5). We were not able to assess effects of timing in the pandemic, since there was hardly any variation in this variable (almost all data were gathered early in the pandemic). There was no consistent evidence for the existence of publication bias. Correcting effect-size estimates and 95% CI’s for the potential effects of publication bias largely yielded similar results and in no case warranted categorical different conclusions (Table 2).

## Discussion

The present meta‐analysis pooled 46 independent studies on a total of 4,688,559 subjects. Age range of the samples in the included studies was 7 to 65 and in a majority of cases, female participants were overrepresented. Data predominantly were gathered in the USA, southern and northern Europe and Australia (41 of 46 samples). Our results show that the prevalence of the EDs and their symptoms increased during the COVID-19 pandemic relative to time frames prior to this. Symptom severity of AN and comorbid depression, but not anxiety and suicidality, were also found to be higher during the pandemic. Concordantly, we found that hospitalization, emergency department visits and in-patient admission rates were found to be higher during the pandemic. We report, with moderate strength of evidence, that in the general population, BE behavior was reported more often in times of the pandemic. We did not observe such increases in EE behavior. With regard to this latter null finding, it should be noted that the results were influenced by an outlier in the data. Freitas et al*.* [53] reported a decrease in EE during the pandemic, relative to prior to the pandemic. This study, however, applied different measurements pre- and during the pandemic (interview versus self-report, respectively), which could have driven the unsuspected results. When we excluded this study from our meta-analysis, EE symptoms were observed to be higher during the Covid-19 pandemic (*p* < 0.01, BF favoring H_1_ = 3.48). There were no consistent associations between the prespecified moderators and there was little evidence for publication bias. We were not able to investigate timing of the pandemic on outcome because there was too little variance in this variable to allow analysis.

This was not a study that investigated mechanisms or mediators, yet we do have some hypotheses that could explain the pattern of results that we obtained. First of all, the pandemic and resulting quarantines, restrictions and social isolation may have increased feelings of stress, ineffectiveness, loss of control, fear and boredom [81–83]. EDs have often been conceptualized as maladaptive responses to stressful situations [84, 85], that could temporarily decrease awareness and intensity of the aversive emotions [54, 86, 87]. Second, the pandemic induced disruptions in daily routines caused changes in usual patterns of physical activity, eating, and sleeping routines [25, 88–90]. This may have brought forth concerns of gaining weight, which is common in people with EDs [25, 91], and increased the symptoms of EDs [90] and related behaviors such as EE. Third, the immense increase in social media usage during the pandemic may have led to raised worries regarding body appearance and disordered eating [92–94]. Fourth, limiting grocery trips and food access may have resulted in food insecurity, worries about the availability of food, and hoarding that are related to urges to binge [12, 27]. A fifth potential explanation of our findings is in term of limited access to professional services and social sources, due to lockdowns [77, 90].

Our findings are largely similar to those reported by previous systematic reviews and meta-analyses [12, 19–27]. Yet our work stands out from them for several reasons. First of all, we report results on a multitude of data for most associations. We also are able to pool data on more specific outcome types (e.g.*,* ED-related hospitalization and EE). Furthermore, our work stands out by applying both classical null hypothesis significance testing and Bayesian approaches to meta-analyses, and this has proved advantages (e.g.*,* in this we can present evidence for the null hypothesis that the severity of general ED symptoms did not increase in patients during the pandemic). Another strength of our work is that article selection and data extraction were performed by three researchers independently. Another strength is that we pooled data across studies on a small variety of standardized assessment instruments. Limitations of our study include, but are not limited to, the observational nature of our input data. Included studies mainly used convenience sampling that for the larger part relied on self-report of the participants with no follow-up data. Future studies could account for the bias that this might have caused. Future meta-analyses also could compare the results of different methods by which samples were recruited for studies on the impact of the pandemic on ED’s. There were certain limitations with the generalizability of the included studies. For instance, the mean percentage of females was high. Another limitation of our work is that we only could marginally look into the effects of timing in the pandemic and its associations with ED-related psychopathology. From all included studies we extracted an effect size reflecting a change in ED-related psychopathology pre versus during the pandemic. This is informative, but does not capture the course and the dynamics of symptom development and alleviation. The data reported Hill et al*.* [95] show this perfectly with large monthly variation in suicidal ideation and behavior within the pandemic.

Future studies should elaborately investigate the moderating factors for the development and worsening of AN and BN symptomology in specific timeframes. Publication bias seemed evident in the analysis on the broad outcome category ‘need for care’. Adjusting for this by means of trim-and-fill methods led to somewhat lower effect size estimates and a wider confidence interval, yet they remained statistically significant. The Bayes Factor also strongly supported the alternative hypothesis of an effect of the pandemic on need for care over the null hypothesis. Finally, our search may have been limited because of the restricted number of search terms. However, some rather broad terms were used (e.g.*,* eating) and we only included about 1.5% of the initial hits. A reviewer suggested using some more specific terms a.o., compulsive exercise. This, we found in a pilot, resulted in significant more initial hits, but not to more eligible articles. Probably, a term as compulsive exercise is too generic, leading to many hits in for the current work irrelevant domains (e.g.*,* obsessive compulsive disorder or physical activity).

The findings reported in this current systematic review have some clinical implications. The prevalence and/or severity of ED symptoms were found to be increased during the COVID-19 period, suggesting the need to develop intervention and prevention programs for EDs. Such methods preferably are available for a broad range of ED-related symptoms and disorders, and accessible via flexible and easy to rollout web-conferencing software. Even more accessible and proven to be useful, also in times of the pandemic, are freely available apps promoting self-monitoring and teaching coping skills [96]. Furthermore, public health policies and intervention programs should be suitable in case of confinement or related circumstances. Future single studies and meta-analyses could investigate this, together with a focus on moderating effects of age, gender and culture.

To the best of our knowledge, this is the first systematic review with classical and Bayesian meta-analysis that assessed the effect of the COVID-19 pandemic on ED psychopathology and severity, ED-related need for care, psychiatric comorbidity, and BE and EE. We show that the COVID-19 pandemic is associated with a wide spread and worrisome negative effect on ED pathology and ED-related need for care.

### What is already known on this subject?

The COVID-19 pandemic is suspected to have had a negative effect on the prevalence rates of, and indicators for, ED psychopathology. What is unknown is the range and the magnitude of this effect.

### What does this study add?

Our data suggest that in the face of the COVID-19 pandemic, ED prevalence rates, their symptoms, psychiatric comorbidity, and need for care increased. The evidential strength on the existence of these associations ranges from anecdotal (e.g.*,* the pandemic related increase in emotional eating in general population samples) to very strong (e.g.*,* the pandemic related increase in ED-related need for care).

## Supplementary Information

Below is the link to the electronic supplementary material.

Supplementary file1 (DOCX 42 KB)

Supplementary file2 (DOC 295 KB)

## Data Availability

The data that we gathered and generated in this project are available for other projects upon reasonable request.
