# Supplementary material for: COVID-19-related changes in eating disorder pathology, emotional and binge eating and need for care: a systematic review with frequentist and Bayesian meta-analyses
Source: Eat Weight Disord. 2023 Feb 20;28(1):19. doi: 10.1007/s40519-023-01547-2 (PMC9941242; doi:10.1007/s40519-023-01547-2)
Supplement: Supplementary file 1 — Supplementary file1 (DOCX 42 KB) [file 40519_2023_1547_MOESM1_ESM.docx]

**Supplementary results**

*Assessment of methodological quality*

The methodological quality of input studies was scored by 2 members of the author team (ÂG, NM, and/or MM) using the *quality assessment tool for cross-sectional studies* that is recommended by the United States National Institutes of Health (US NIH 2021; <https://www.nhlbi.nih.gov/health-topics/study-quality-assessment-tools>). The items of this tool are provided below, in **Table S2**.

The inter-rater reliability of the quality assessments was high (κ = 0.922, SE = 0.02) (McHugh, 2012).

**Table S1**. Items of the quality assessment tool for cross-sectional studies

| Item |  |
| --- | --- |
| 1 | Was the research question or objective in this paper clearly stated? |
| 2 | Was the study population clearly specified and defined? |
| 3 | Was the participation rate of eligible persons at least 50%? |
| 4 a | Were all the subjects selected or recruited from the same or similar populations (including the same time period)? |
| 4 b | Were inclusion and exclusion criteria for being in the study prespecified and applied uniformly to all participants? |
| 5 | Was a sample size justification, power description, or variance and effect estimates provided? |
| 6 | For the analyses in this paper, were the exposure(s) of interest measured prior to the outcome(s) being measured? |
| 7 | Was the timeframe sufficient so that one could reasonably expect to see an association between exposure and outcome if it existed? |
| 8 | For exposures that can vary in amount or level, did the study examine different levels of the exposure as related to the outcome (e.g., categories of exposure, or exposure measured as continuous variable)? |
| 9 | Were the exposure measures (independent variables) clearly defined, valid, reliable, and implemented consistently across all study participants? |
| 10 | Was the exposure(s) assessed more than once over time? |
| 11 | Were the outcome measures (dependent variables) clearly defined, valid, reliable, and implemented consistently across all study participants? |
| 12 | Were the outcome assessors blinded to the exposure status of participants? |
| 13 | Was loss to follow-up after baseline 20% or less? |
| 14 | Were key potential confounding variables measured and adjusted statistically for their impact on the relationship between exposure(s) and outcome(s)? |

**Table S2**. Reasons for exclusion in the second round of article selection

| **Author** | **Reason** |
| --- | --- |
| Abduljalil Bakhsh et al 2021 | Eating behaviour / dietary adherence |
| Agostino et al 2021 | No useable outcome data |
| Aguilar, 2022 | Eating behaviour / dietary adherence |
| Akgül et al 2022 | No useable outcome data |
| Alafif et al. 2021 | Eating behaviour / dietary adherence |
| Al-Musharaf et al 2020 | No comparision with before the pandemic |
| Asch et al 2021 | No useable outcome data |
| Barcın-Güzeldere et al 2021 | No comparision with before the pandemic |
| Barone et al. 2021 | No useable outcome data |
| Biçer et al 2021 | Eating behaviour / dietary adherence |
| Biemanian et al. 2020 | Eating behaviour / dietary adherence |
| Bialec-Dratwa et al. 2022 | Eating behaviour / dietary adherence |
| Birgegård et al 2022 | No comparision with before the pandemic |
| Buckley et al. 2021 | Eating behaviour / dietary adherence |
| Cascino et al 2021 | Contacted the author for data, no response |
| Cecchetto et al 2021 | No comparision with before the pandemic |
| Cerniglia and Cimino, 2022 | No useable outcome data |
| Chadi et al 2021 | Contacted the author for data, no response |
| Chan & Chiu 2020 | Eating behaviour / dietary adherence |
| Christensen et al. 2020 | No useable outcome data |
| Clark Bryan et al. 2020 | Qualitive data |
| Coimbra et al. 2022 | Eating behaviour / dietary adherence |
| Costa et al. 2022 | Eating behaviour / dietary adherence |
| Coulthard et al. 2021 | Eating behaviour / dietary adherence |
| Cui et al 2021 | Eating behaviour / dietary adherence |
| Cross and Morris 2021 | Contacted the author for data, the data is not suitable. |
| Czepczor-Bernat et al. 2021 | No comparision with before the pandemic |
| Daly and Costigan, 2022 | No useable outcome data |
| Daniel et al 2022 | Eating behaviour / dietary adherence |
| Davies et al 2021 | No useable outcome data |
| Dey et al 2022 | Perspective |
| Dhemaid et al. 2021 | No comparision with before the pandemic |
| D. Santos Quaresma et al. 2021 | No comparision with before the pandemic |
| Eyupoglu et al 2022 | Eating behaviour / dietary adherence |
| Feinmann, 2021 | No useable outcome data |
| Fernandez-Aranda et al. 2020 | No comparision with before the pandemic |
| Flaudias et al. 2020 | Eating behaviour / dietary adherence |
| Frayn et al. 2021 | No comparision with before the pandemic |
| Freizinger et al. 2022 | No comparision with before the pandemic |
| Gao et al. 2021 | No comparision with before the pandemic |
| Gao et al. 2022 | Review/meta-analysis |
| Gillion-Keren et al. 2022 | Eating behaviour / dietary adherence |
| Guerrini Usubini et al 2021 | No comparision with before the pandemic |
| Guzek et al. 2021 | Eating behaviour / dietary adherence |
| Hadar-Shoval et al. 2021 | Eating behaviour / dietary adherence |
| Hadar-Shoval et al. 2022 | No comparison with before the pandemic |
| Haghshomar et al. 2022 | Review/meta-analysis |
| Hartman-Munick et al. 2022 | No useable outcome data |
| Hill et al.2022 | No useable outcome data |
| Hudson et al 2022 | No useable outcome data |
| Jackson et al. 2022 | Eating behaviour / dietary adherence |
| Jordan et al 2021 | Contacted the author for data, did not hear back. |
| Joseph Louis et al 2021 | No comparison with before the pandemic |
| Katie et al 2022 | No useable outcome data |
| Katzman, 2021 | Not a research article |
| Kim et al. 2021 | No comparison with before the pandemic |
| Kurisu et al. 2022 | No useable outcome data |
| Leenaerts et al. 2021 | No comparison with before the pandemic |
| Lin et al 2021 | Contacted the author for data, no response |
| Linardon et al. 2021 | Review/meta-analysis |
| Lo Moro et al. 2022 | No comparison with before the pandemic |
| López-Moreno et al. 2020 | Eating behaviour / dietary adherence |
| Lurie et al. 2021 | No useable outcome data |
| Madan et al. 2021 | Eating behaviour / dietary adherence |
| Mahar et al. 2021 | No useable outcome data |
| Mansfield et al. 2021 | No comparison with before the pandemic |
| Martínez-Vázquez et al 2021 | Eating behaviour / dietary adherence |
| Matinez-Rodrigez et al. 2021 | Eating behaviour / dietary adherence |
| Matthews et al. 2021 | No useable outcome data |
| McAtamney et al. 2021 | Eating behaviour / dietary adherence |
| McLean et al. 2022 | Systematic review |
| McDonnel et al. 2022 | No comparison with before the pandemic |
| McNicholas & Moore 2022 | No useable outcome data |
| Meda et al. 2021 | No useable outcome data |
| Meier et al. 2022 | Review |
| Mehta et al. 2021 | Review |
| Modrzejewska et al. 2021 | Eating behaviour / dietary adherence |
| Mohapatra et al. 2022 | No comparison with before the pandemic |
| Monteleone et al. 2021 | No useable outcome data |
| Monteleone et al 2021 (b) | No useable outcome data |
| Monteleone et al 2021(c) | No useable outcome data |
| Monteleone et al 2021(d) | No useable outcome data |
| Mumtaz et al 2022 | Review |
| Muth et al. 2022 | No comparison with before the pandemic |
| Muzi et al. 2021 | No useable outcome data |
| Nisticó et al. 2021 | No comparison with before the pandemic |
| Otto et al. 2021 | No useable outcome data |
| Ozen et al. 2021 | Eating behaviour / dietary adherence |
| Phillipou et al. 2020 | Eating behaviour / dietary adherence |
| Phillipou et al 2021 | Contacted the author for data, no response |
| Pineda-Gacia et al. 2021 | Eating behaviour / dietary adherence |
| Qingqing et al. 2021 | Eating behaviour / dietary adherence |
| Quittkat et al. 2020 | No useable outcome data |
| Ramalho et al 2022 | No comparison with before the pandemic |
| Revet et al 2021 | No useable outcome data |
| Reed and Oort 2021 | Contacted the author for data, did not hear back. |
| Rossi et al 2021 | No useable outcome data |
| Sagribay III et al. 2022 | Only includes behaviour not disorder |
| Sadler et al. 2021 | Eating behaviour / dietary adherence |
| Scacchi et al. 2021 | Eating behaviour / dietary adherence |
| Schulte et al. 2022 | Eating behaviour / dietary adherence |
| Schwartz et al. 2021 | Contacted the author for data, but no info received |
| Sikaroudi et al. 2021 | No comparision with before the pandemic |
| Silverman et al 2021 | Eating behaviour / dietary adherence |
| Skolmowska et al. 2022 | Eating behaviour / dietary adherence |
| Solmi et al. 2021 | No useable outcome data |
| Spigel et al. 2021 | Eating behaviour / dietary adherence |
| Takakura et al 2022 | No comparision with before the pandemic |
| Tavolacci et al. 2021 | No comparision with before the pandemic |
| Tavolacci et al 2021 (b) | No useable outcome data |
| Tfifha et al. 2021 | No comparision with before the pandemic |
| Thompson et al. 2021 | No useable outcome data |
| Troncone et al. 2020 | Eating behaviour / dietary adherence |
| Vacca et al 2021 | No comparision with before the pandemic |
| Vall Roque et al. 2021 | No useable outcome data |
| Vall-Roqué et al 2021 (b) | No comparision with before the pandemic |
| Vitagliano et al. 2021 | No useable outcome data |
| Vyer & Katzman. 2021 | No useable outcome data |
| Wang et al 2021 | Eating behaviour / dietary adherence |
| Warne et al. 2021 | Eating behaviour / dietary adherence |
| Zeigler, Z 2021 | No useable outcome data |
| Zipfel et al 2022 | No useable outcome data |

**Table S3.** *Overview of the reported instruments used to assess AN, BN, BED and ED’s in General*

| Authors | Instrument used | |
| --- | --- | --- |
| Athanasiadis et al. (2021) | ELOCS^bc^ | |
| Baenas et al. (2021) | CIES^abc^ | |
| Bianchi et al. (2022) | BEDS-7 | |
| Breiner et al. (2021) | EDE-Q^abcd^ | |
| Castellini et al. (2020) | EDE-Q^abc^ | |
| Elmacioglu et al. (2020) | TFEQ-R18^e^ | |
| Freitas et al. (2021) | TFEQ-R21^e^ | |
| Giel et al, (2021) | EDE^bcd^ | |
| Machado et al. (2020) | EDE-Q^abc^ , CIA^c^ | |
| Martinez de Quel et al. (2021) | EAT-26^abc^ | |
| Miskovic-Wheatly et al. (2022) | EDE-Q^ac^ | |
| Özcan and Yeşikaya (2021) | EEQ-TR^e^ | |
| Schlegl et al. (2020) a | SDQ^acd^ | |
| Schlegl et al. (2020) b | SDQ^bce^ | |
| Spettigue et al. (2021) | EDQ-A^abcd^ | |
| Tazeoglu et al. (2021) | DEBQ^ae^ | |
| Temorshuizen et al. (2020) | EDI^bcd^ | |
| Trott et al. (2021) | EAT-26^abc^, EAI^bc^, BDDQ^c^ | |
| Abbreviations. BDDQ, Body Dysmorphic Disorder Questionnaire, BEDS-7 = Binge Eating Disorder. Screener-7, chEAT = Children’s eating Attitudes Test, CIES = COVID-19 Isolation Scale, EAI = Exercise Addiction Inventory, EAT-26 = Eating Attitude Test-26, EDE = Eating Disorder Examination, EDE - Q = Eating Disorder Examination Questionnaire, EDEQ-A = Eating Disorder Examination Questionnaire for Adolescents, EDI-2 = Eating Disorder Inventory 2, EEQ-TR = Emotional Eater Questionnaire-Turkish, ELOCS = Eating Loss of Control Scale, DEBQ = Dutch Eating Behavior Questionnaire, SDQ = Self Developed, TFEQ-R18 = Three Factor Nutrition Questionnaire, TFEQ-R2 1=The 21-item Three-Factor Eating Questionnaire  ^a^ used to assess anorexia  ^b^ used to assess bulimia  ^c^ used to assess general eating disorder  ^d^ used to assess binge eating disorder  ^e^ emotional eating behaviour | |  |

**Table S4.** Mental health disorder and COVID-19 course variables from Bayesian meta-analysis

| ***ED patient data*** |  |  | **Evidence for H_0_** | **Evidence for H_1_** | |
| --- | --- | --- | --- | --- | --- |
|  | *k* | *N* | BF_01_ [prior = 0.0] | BF_10_ [prior = 0.0] | Posterior probability |
| ***Prevalence rates ED*** |  |  |  |  |  |
| AN / MIX | 10 | 33032 | 0.14 | 7317 | 0.88 |
| ***ED symptom prevalence*** |  |  |  |  |  |
| AN increase symptom prevalence | 5 | 1394 | 0.52 | 2504 | 0.72 |
| BN increase symptom prevalence | 6 | 1346 | 0.44 | 2263 | 0.69 |
| ***ED symptom severity*** |  |  |  |  |  |
| ED severity score | 11 | 1875 | 1.72 | 0.42 | 0.30 |
| AN severity | 7 | 3095 | 0.04 | 23.17 | 0.95 |
| BN severity | 7 | 2873 | 0.21 | 4.72 | 0.82 |
| BED severity | 4 | 2243 | 0.43 | 1.71 | 0.63 |
| ***Comorbid psychiatric symptoms*** |  |  |  |  |  |
| Depression | 10 | 2012 | 0.15 | 6.85 | 0.86 |
| Anxiety | 9 | 1970 | 0.21 | 4.86 | 0.83 |
| Suicidality | 3 | 8722 | 1.40 | 0.71 | 0.42 |
| ***Need for care and impairment*** |  |  |  |  |  |
| Mix category ^2^ | 13 | 5260823 | 0.0001 | 9799201 | 1.00 |
| Hospitalization | 4 | 4862668 | 0.013 | 75849 | 0.99 |
| Length of stay | 5 | 1099 | 1.13 | 0.88 | 0.47 |
| Age at admission | 5 | 837 | 0.76 | 2.60 | 0.58 |
| ***General population data*** |  |  |  |  |  |
| ***ED related behaviors*** |  |  |  |  |  |
| Binge eating | 8 | 4480 | 0.08 | 9.18 | 0.90 |
| Emotional eating | 6 | 1091 | 0.35 | 2.89 | 0.77 |

*Abbreviations*. AN, Anorexia Nervosa; BF, Bayes Factor; BED, Binge Eating Disorder; BN, Bulimia Nervosa; ED, Eating Disorder; *k*, number of effect-size estimates; *N*, number of participants.

**Table S5.** Results from meta-regression analyses

| ***ED patient data*** |  |  |  |
| --- | --- | --- | --- |
|  | Percentage women | Average age | Methodological quality |
| *Prevalence rates ED* |  |  |  |
| All / mix | -0.05 (SE = 0.06) | -0.007 (SE = 0.01) | 0.14 (SE = 0.06) * |
| *ED symptom prevalence* |  |  |  |
| AN increase symptom prevalence | .003 (SE = 0.03) | .03 (SE = 0.01) * | 0.05 (SE = 0.10) |
| BN increase symptom prevalence | .001 (SE = 0.02) | .02 (SE = 0.01) * | 0.02 (SE = 0.07) |
| AN decrease symptom prevalence | Not sufficient data | Not sufficient data | Not sufficient data |
| BN decrease symptom prevalence | 0.07 (SE = 0.05) | -0.01 (SE = 0.01) | -0.04 (SE = 0.06) |
| *ED symptom severity* |  |  |  |
| ED severity general | 0.003 (SE = 0.009) | 0.03 (SE = 0.02) | 0.02 (SE = 0.07) |
| AN severity | .01 (SE = 0.006) * | .004 (SE = 0.02) | - .01 (SE = 0.03) |
| BN severity | .02 (SE = 0.007) ** | .01 (SE = 0.02) | -.02 (SE = 0.03) |
| BED severity | Not sufficient data | Not sufficient data | Not sufficient data |
| *Comorbid psychiatric symptoms* |  |  |  |
| *Depression* | 0.002 (SE = 0.005) | 0.006 (SE = 0.006) | 0.02 (SE = 0.04) |
| *Anxiety* | -0.01 (SE = 0.03) | -0.05 (SE = 0.04) | 0.14 (SE = 0.36) |
| *Suicidality* | Not sufficient data | Not sufficient data | Not sufficient data |
| *Need for care and impairment* |  |  |  |
| Mix category ^2^ | 0.007 (SE = 0.006) | 0.05 (SE = 0.03) | -0.12 (SE = 0.07) |
| Hospitalization | Not sufficient data | Not sufficient data | Not sufficient data |
| Length of stay | Not sufficient data | Not sufficient data | Not sufficient data |
| Age at admission | Not sufficient data | Not sufficient data | Not sufficient data |
| ***General population data*** |  |  |  |
|  | Percentage women | Average age | Methodological quality |
| *ED related behaviors* |  |  |  |
| Binge eating | -0.004 (SE = 0.01) | 0.003 (SE = 0.02) | -0.03 (SE = 0.10) |
| Emotional eating | 0.009 (SE = 0.04) | 0.03 (SE = 0.06) | -0.35 (SE = 0.15) * |

* *P* < .05, ** *P* < .01, *** *P* < .001

*Abbreviations*. AN, Anorexia Nervosa; BED, Binge Eating Disorder; BN, Bulimia Nervosa; ED, Eating Disorder; SE, Standard Error.

**5.**
