# Supplementary material for: COVID-19-related changes in eating disorder pathology, emotional and binge eating and need for care: a systematic review with frequentist and Bayesian meta-analyses
Source: Eat Weight Disord. 2023 Feb 20;28(1):19. doi: 10.1007/s40519-023-01547-2 (PMC9941242; doi:10.1007/s40519-023-01547-2)
Supplement: Supplementary file 2 — Supplementary file2 (DOC 295 KB) [file 40519_2023_1547_MOESM2_ESM.doc]

| **Section/topic** | **#** | **Checklist item** | **Reported on page ^1^** |
| --- | --- | --- | --- |
| **TITLE** | | |  |
| Title | 1 | Identify the report as a systematic review, meta-analysis, or both. | 3 |
| **ABSTRACT** | | |  |
| Structured summary | 2 | Provide a structured summary including, as applicable: background; objectives; data sources; study eligibility criteria, participants, and interventions; study appraisal and synthesis methods; results; limitations; conclusions and implications of key findings; systematic review registration number. | 4 |
| **INTRODUCTION** | | |  |
| Rationale | 3 | Describe the rationale for the review in the context of what is already known. | 5 |
| Objectives | 4 | Provide an explicit statement of questions being addressed with reference to participants, interventions, comparisons, outcomes, and study design (PICOS). | 5 |
| **METHODS** | | |  |
| Protocol and registration | 5 | Indicate if a review protocol exists, if and where it can be accessed (e.g., Web address), and, if available, provide registration information including registration number. | 6 |
| Eligibility criteria | 6 | Specify study characteristics (e.g., PICOS, length of follow-up) and report characteristics (e.g., years considered, language, publication status) used as criteria for eligibility, giving rationale. | 6 |
| Information sources | 7 | Describe all information sources (e.g., databases with dates of coverage, contact with study authors to identify additional studies) in the search and date last searched. | 6 |
| Search | 8 | Present full electronic search strategy for at least one database, including any limits used, such that it could be repeated. | 6 |
| Study selection | 9 | State the process for selecting studies (i.e., screening, eligibility, included in systematic review, and, if applicable, included in the meta-analysis). | 7 |
| Data collection process | 10 | Describe method of data extraction from reports (e.g., piloted forms, independently, in duplicate) and any processes for obtaining and confirming data from investigators. | 7 |
| Data items | 11 | List and define all variables for which data were sought (e.g., PICOS, funding sources) and any assumptions and simplifications made. | 7 |
| Risk of bias in individual studies | 12 | Describe methods used for assessing risk of bias of individual studies (including specification of whether this was done at the study or outcome level), and how this information is to be used in any data synthesis. | 7 |
| Summary measures | 13 | State the principal summary measures (e.g., risk ratio, difference in means). | 8 |
| Synthesis of results | 14 | Describe the methods of handling data and combining results of studies, if done, including measures of consistency (e.g., I^2^) for each meta-analysis. | 8 |

Page 1 of 2

| **Section/topic** | **#** | **Checklist item** | **Reported on page #** |
| --- | --- | --- | --- |
| Risk of bias across studies | 15 | Specify any assessment of risk of bias that may affect the cumulative evidence (e.g., publication bias, selective reporting within studies). | 7 and esuppl |
| Additional analyses | 16 | Describe methods of additional analyses (e.g., sensitivity or subgroup analyses, meta-regression), if done, indicating which were pre-specified. | 8 |
| **RESULTS** | | |  |
| Study selection | 17 | Give numbers of studies screened, assessed for eligibility, and included in the review, with reasons for exclusions at each stage, ideally with a flow diagram. | 8 |
| Study characteristics | 18 | For each study, present characteristics for which data were extracted (e.g., study size, PICOS, follow-up period) and provide the citations. | 8-9 |
| Risk of bias within studies | 19 | Present data on risk of bias of each study and, if available, any outcome level assessment (see item 12). | 9 |
| Results of individual studies | 20 | For all outcomes considered (benefits or harms), present, for each study: (a) simple summary data for each intervention group (b) effect estimates and confidence intervals, ideally with a forest plot. | 9 and supple |
| Synthesis of results | 21 | Present results of each meta-analysis done, including confidence intervals and measures of consistency. | 11-12 |
| Risk of bias across studies | 22 | Present results of any assessment of risk of bias across studies (see Item 15). | 12 |
| Additional analysis | 23 | Give results of additional analyses, if done (e.g., sensitivity or subgroup analyses, meta-regression [see Item 16]). | 11-12 |
| **DISCUSSION** | | |  |
| Summary of evidence | 24 | Summarize the main findings including the strength of evidence for each main outcome; consider their relevance to key groups (e.g., healthcare providers, users, and policy makers). | 13 |
| Limitations | 25 | Discuss limitations at study and outcome level (e.g., risk of bias), and at review-level (e.g., incomplete retrieval of identified research, reporting bias). | 13-14 |
| Conclusions | 26 | Provide a general interpretation of the results in the context of other evidence, and implications for future research. | 15 |
| **FUNDING** | | |  |
| Funding | 27 | Describe sources of funding for the systematic review and other support (e.g., supply of data); role of funders for the systematic review. | 1 |

^1^ location is based on unedited manuscript that we report below

*From:*  Moher D, Liberati A, Tetzlaff J, Altman DG, The PRISMA Group (2009). Preferred Reporting Items for Systematic Reviews and Meta-Analyses: The PRISMA Statement. PLoS Med 6(7): e1000097. doi:10.1371/journal.pmed1000097

For more information, visit: **www.prisma-statement.org**.

**COVID-19 related changes in eating disorder pathology, emotional and binge eating and need for care: a systematic review with frequentist and Bayesian meta-analyses**

Âmine Güzel ^1^, Naz Lâl Mutlu ^1^, Marc Molendijk ^1,2^

*Affiliations*

^1^ Clinical Psychology Department, Leiden University, Leiden, The Netherlands

^2^ Leiden Institute of Brain and Cognition, Leiden University Medical Center, Leiden, The Netherlands

*Correspondence*

Marc Molendijk, PhD
*E-mail addresses:* [m.l.molendijk@fsw.leidenuniv.nl](mailto:m.l.molendijk@fsw.leidenuniv.nl)

*Orchid:* <https://orcid.org/0000-0002-2204-9361>

*Author contributions*

Concept and design: all authors.

Drafting of the manuscript: all authors.

Critical revision of the manuscript for important intellectual content: all authors.

Âmine Güzel and Naz Lâl Mutlu contributed equally to this study

*Funding*

There was no funding for this project

*Declaration of interest*

The authors declare no competing interests.

*Acknowledgements*

We thank dr Dawn Branley-Bell, dr David Asch, dr Cynthia Bulik and dr Matthijs Kruizinga for sharing additional data upon request. We are grateful to our colleagues at Leiden University for their helpful comments and the discussions on the subject matter.

*Manuscript information*

Number of words abstract: 243

Number of words main body: 3.698

Number of figures and tables: 1 figure, 2 tables

Number of references: 96

Number of pages: 18

Supplementary materials: 2 files (PRISMA Checklist) and supplementary methods and results

**Abstract**

*Purpose*

The COVID-19 pandemic has been a leading cause of stress and feelings of loss of control, both of which have been related to Eating Disorder (ED) pathology onset and deterioration. We aim to estimate the magnitude of changes in the prevalence rates of, and indicators for, ED psychopathology in the face of the COVID-19 pandemic.

*Method*

Pre-registered systematic review with frequentist and Bayesian meta-analyses. Searches for eligible studies were performed in PubMed, Web of Science and pre-print servers until January 15 2023.

*Results*

Our searches yielded 46 eligible studies reporting on a total of 4.688.559 subjects. These data provide strong evidence indicating increased rates of diagnosed and self-reported ED’s and a concordant increased need for care in the face of the pandemic. ED symptom severity scores in patients were not elevated during the pandemic, except for those related to anorexia nervosa. On average, people in the general population report relatively high levels of emotional and binge eating during the pandemic, although the evidential strength for these associations is only anecdotal to moderate. Moderators of between-study heterogeneity were not detected.

*Conclusions*

Altogether, our results suggest that the COVID-19 pandemic is associated with a wide spread negative effect on ED pathology in patient samples and the general population. The development of online prevention and intervention programs for EDs during stressful times like a pandemic is encouraged. A limitation is that the results reported here may be prone to biases, amongst others, self-report bias.

*Level of evidence*

Level I, systematic review and meta-analysis

*Preregistration*

Prospero [https://www.crd.york.ac.uk/prospero] ID: CRD42022316105

*Keywords*

Eating disorders, Anorexia Nervosa, Bulimia Nervosa, Binge Eating, Emotional Eating, COVID-19, Corona, Lockdown

**Introduction**

In December 2019, the SARS‑CoV‑2 abruptly spread worldwide, causing the coronavirus disease (Covid-19) pandemic [1, 2]. This pandemic has proven to be a huge source of stress and caused a profound disruption in millions of people's life for multiple reasons (World Health Organization, 2022 [3]). A worsening in mental health symptoms and increased rates of psychopathology has been observed following the start of the pandemic in children, adolescents, and adults [4-6].

The onset of Eating Disorders (ED) and related behaviors such as Binge Eating (BE) and Emotional Eating (EE) in general population and at-risk samples has been related to stress exposure and adversity [7-9]. Theories even suggest that pathological eating behaviors can serve as a coping response to deal with stress and adverse emotions [10, 11]. Given the stress generating nature of the Covid-19 pandemic and its profound impact on emotional wellbeing, alternations in the prevalence and severity of EDs and related behaviors are to be expected, together with a concordant increase in the need for ED related care. Indeed, several studies show such trends. At the diagnostic level, studies exist that report increases in the rates of Anorexia Nervosa (AN) and Bulimia Nervosa (BN)[12, 13]. A worsening of symptoms of individuals with ED’s and higher rates of BE and EE has been reported as well in people with AN and BN [14, 15]. In line with this are findings of an increased demand for care related to ED, such as an increased hospitalization rate due to AN [16-18].

We know of five systematic reviews on the topic [12, 19-23]. Five of them [12, 20-23] sketch the picture that ED pathology worsened in the general population and in AN and BN patient populations and that related care demand increased with the advent of the pandemic. One of the reviews, however, concludes that the evidence for Covid-19’s impact on ED psychopathology in the general population and in patients is limited due to methodological weaknesses of the available data [19]. To our knowledge, four quantitative meta-analyses were performed on this topic [24-27]. Sidelli *et al.* [24] found that about 50% of people with an ED (AN, BN, or BED) reported a worsening of symptoms in relation to the pandemic. Similar findings, yet based on a larger database, were reported by Haghshomar *et al.* [25]. Khraisat *et al.* [26] also reports an overall increase in ED symptoms, and beyond that highlights a significant increase in comorbid anxiety and depression symptoms in people with an ED diagnosis. Based on 11 studies, Gao *et al.* [27] report an overall symptom deterioration. Associated factors, amongst which anxiety and social isolation, are also reported on by these authors.

Many relevant studies have been published since the publication of the excellent meta-analyses, specified above and hence an update seems relevant. In an updated meta-analysis, we see fit for the assessment of effects of timing of the pandemic (*i.e.,* whether observations are similar in the early phase of the pandemic, relative to later phases). Assessment on pandemic related changes in prevalence rates and symptom severity of specifically AN, BN and BED and of changes in BE and EE also seem relevant add-ons. Finally, we want to sketch a broad picture of the pandemic effects on ED patients and hence we will also meta-analyze its effects on comorbid mental health syndromes and symptoms (*e.g.,* suicidality) and on the use of care by ED patients (*e.g.,* emergency room visits for ED symptoms). Finally, we aim to summarize the existing data on changes in EE and BE in the general population in the face of the pandemic. We find this relevant in order to shed some further light on the continuum of eating behaviors and EDs [28].

Overall, we hypothesize a wide spread negative effect of the pandemic on ED related pathology, related behaviors, need for care, and psychiatric comorbidity. We will present findings from both classical (*i.e.,* frequentist) and Bayesian meta-analysis because these approaches complement each other in achieving our goals. The classical meta-analyses will provide the option for significance testing of the null hypothesis (*i.e.,* the hypothesis that the pandemic had no effect on outcome). The Bayesian approach will allow us to discern whether there is “evidence of absence of an effect” versus “absence of evidence”, that is whether the data is inconclusive or whether there is evidence suggesting no effect of the pandemic on the outcomes of interest [29]. The knowledge that we will generate may contribute to theory on the origins of pathological eating behaviors and could inform policy regarding the development of prevention and intervention programs.

**Method**

Our method and approach were carried out in compliance with the Meta-analysis Of Observational Studies in Epidemiology (MOOSE) group [30] and PRISMA guidelines [31]. The PRISMA checklist for this study is presented as supplementary material. This meta-analysis has been pre-registered In the International Prospective Register of Systematic Reviews (PROSPERO, ID CRD42022316105).

**Search and Selection Strategy**

PubMed and Web of Science were systematically searched for eligible studies. Systematic searches were supplemented with a non-systematic search in Google Scholar. A grey literature search on the preprint servers Psyrxiv.org and Biorxiv.org was performed. Data bases were searched from their inception. Official searches started March 19 2022 and final search date was January 15, 2023. The following search terms were used; ((Covid* OR Corona OR SarS* OR Lock down) AND ("emotional eating" OR binge* OR eating OR Bulim* OR Anorex*)). The researchers independently conducted the literature search. After screening and first selection, full text versions of the articles were evaluated to come to a decision on final inclusion. We applied the inclusion and exclusion criteria (see below) in making this decision. Cases of disagreement were resolved through discussion and consensus.

**Inclusion and exclusion criteria**

Articles were included when they (1) were case-control studies, retrospective cohort studies, and prospective cohort studies, (2) were written in English, Dutch, Spanish, Turkish, German, or French, (3) reported on changes in ED pathology and related behaviors in humans of all ages (see below under *data extraction*) pre versus during the Covid-19 pandemic or at several time points within the pandemic. Articles were excluded when they were not based on original data (*e.g.,* reviews). When data related to our study was missing where it was expected that such data was gathered in a study, the data was requested by contacting the corresponding author of that particular study. In case data could not be obtained after this request, the study was excluded. This occurred in four instances (see **Table S1**).

**Data extraction**

Study selection and data extraction were performed independently by all authors. We extracted data on the total number of participants, year and month of data gathering, demographic information (mean age in years, gender distribution, the country in which the study was performed), clinical information (method of diagnostic assessment, type of disorder, and the presence of symptoms at baseline and follow-up), and outcome data (raw numbers or effect-size estimates and their corresponding 95% confidence interval. For patient samples these include the following outcome variables: (I) prevalence rates of AN, BN, and BED as assessed pre- and during the pandemic, (II) cross sectional data on self-reported changes in ED symptomatology (*e.g.,* pre-clinical symptoms emerged/disappeared in the face of the pandemic), (III) questionnaire data on changes in the severity of ED symptoms as assessed pre- and during the pandemic, (IV) comorbid depression, anxiety and suicidality assessed by different means pre- and during the pandemic, (V) need for care and access to care, including age at admission and duration of admission (*e.g.,* number of hospital days) and clinical impairment pre- versus during the pandemic. For non-patient samples we extracted (VI) questionnaire data on changes in the frequency and intensity of EE and BE assessed pre- versus during the pandemic.

**Assessment of Methodological Quality**

Retained articles were assessed on methodological quality. This was done independently by all authors using the method proposed by Lievense *et al.* [32]. The items that compose this unidimensional scale are presented in **Table S2**. Methodological quality of the articles did not serve as in or exclusion criterion. Rather, continuous methodological quality scores were related to outcomes in moderator analyses.

**Statistical Analysis**

Meta-analyses were conducted in Stata version 17 [33] and JASP. Summary tables on the characteristics of included articles were created. Separate random-effects meta-analyses [34] were conducted on the 6 outcomes reported under data extraction (I-VI). In case a meta-analysis was conducted on different disorder types, *e.g.,* AN and BED, sensitivity analyses were performed. When there was sufficient data, we also investigated differences in outcome during the pandemic, defined as first year *versus* second year. Outcomes were reported as weighted and pooled odds ratios (OR) in case of categorical outcome variables, Cohen’s *d* for continuous outcome variables, and weighted proportions for proportional data. All outcomes were reported with corresponding 95% confidence intervals (CI). Between-study heterogeneity was expressed as I² and tested for statistical significance using the Q statistic asymmetry [35]. Statistical significance was set at *P* < 0.05. We repeated all analyses using a Bayesian approach. From these analyses we reported the results from the random effects model and the averaged Bayes Factor (BF_10_) for priors of *d* = 0 or Odds Ratio (OR) = 1 and with scale parameter 0.5 as main outcome. For one-sided hypothesis testing, we truncated values below 0 for Cohen’s d and below 1 for OR’s. BF’s were interpreted according to the suggestions provided by Morey and Rouder [36]. Moderator analyses were performed in case of statistically significant between-study heterogeneity on outcomes that were based on > 5 independent studies. The tested moderators were: (I) gender, operationalized as percentage women in a given sample, (II) age operationalized as adolescent samples *versus* adult samples, and (III) methodological quality. Gender and age were assessed as effect moderators because these variables are strongly related to variance in ED onset and course [37, 38]. Methodological quality was related to outcome since variance in this variable can be related to effect size estimates, maybe notably so in COVID-19 related studies [39]. Estimates of publication bias were generated by means of Egger's regression test for the assessment of funnel plot asymmetry [35].

**Results**

Overall, 2446 records were identified after the removal of duplicates. After screening these records, 149 records were assessed in full text for eligibility. Interrater agreement on first selection was high (Cohens Kappa = 0.74; SE = 0.05). Based on a comment by a reviewer we reran the search with the term OSFED included. This did not lead to additional hits. **Figure 1** shows the flowchart and outlines the information on the identification, screening, and inclusion of record. A total of 46 independent studies were included in the review, reporting on a total of 4.688.559 subjects.

Records identified through databases PUBMED and Web of Science (*k* = 4.655). Records identified through reference lists and grey literature (*k* = 13).

Total identified records (*k*) = 4.668

Identification

Screening

Records excluded (*k* = 2.981)

Records screened after duplicates removed (*k* = 3.149)

Records excluded, with reasons (*k* = 122)
- No pre- vs pandemic comparision (*k* = 29)

- Contact missing data, no response (*k* = 8)

- No original data (*k* = 10)

- Dietary behavior (*k* = 35)

- Other (*k* = 40)

See the online supplement for further details

Full-text assessment (*k* = 168)

Eligibility

Total *K* = 46 independent studies (*N*_summed_ = 4.688.559)

.

Included

**Figure 1.** Flowchart on identification, screening and inclusion of eligible publications

Average ages of the included samples ranged from 7 to 65 years (average age per sample = 23 years). Women were overrepresented in 42 of the 46 included studies (average percentage of women = 80%). Data were gathered in 16 different countries. Western countries yielded most input samples (*i.e.,* 7 from the USA, 6 from Germany, and 6 from Italy). There was no eligible data from Asia (except from Turkey), Africa, or South America. Key characteristics of the included studies are presented in **Table 1**. Reasons for exclusion in the second round of article selection is provided in **Table S1**. Methods with which symptom severity was assessed are presented in **Table S2**.

**Table 1.** Characteristics of included samples

| **Study** | **ED** | **Age ^1^** | **% female** | **Country** | ***N*** | **Meta-analysis** |
| --- | --- | --- | --- | --- | --- | --- |
| Albert *et al.* 2021 [16] | BE | 48 | 68 | Italy | 56 | II, VI |
| Allgaier *et al.* 2022 [40] | MIX | 14 | N/K | Germany | 47 | V |
| Baenas *et al.* 2021 [14] | AN, BN | 28 | 70 | Mix | 829 | II, IV |
| Branley-Bell & Talbot 2020 [41] | AN, BN | 16-65 | 94 | UK | 129 | III |
| Bianchi *et al.,* 2022 [42] | BE | 24 | 72 | Italy | 1925 | VI |
| Breiner *et al.* 2021 [43] | BE | 28 | 91 | USA | 159 | VI |
| Carison *et al.* 2022 [44] | ED | 7-18 | 72 | Australia | 30170 | V |
| Castellini *et al.* 2020 [45] | AN, BN | 31 | 100 | Italy | 171 | II, VI |
| Christensen *et al.* 2020 [46] | AN, BN | 21 | 75 | US | 579 | I |
| Colleluori *et al.* 2021 [47] | AN, BN | N/K | N/K | Italy | 453 | III |
| Datta *et al.* 2022 [48] | AN | 15 | 96 | Australia | 50 | II, IV |
| Elmacioglu et al., 2020 [49] | EE | 33 | 80 | Turkey | 1036 | VI |
| Eray and Sahin 2021 [50] | MIX | 14 | 57 | Turkey | 272 | I |
| Favreau et al., 2021 [51] | AN, BN | NK | 70 | Germany | 113 | III |
| Feldman et al., 2022 [52] | MIX | 14 | 71 | USA | 71 | IV |
| Freitas et al., 2021 [53] | None | 21 | 100 | Brasil | 71 | VI |
| Giel *et al.* 2021 [54] | MIX | 42 | 81 | Germany | 34 | II, IV |
| Goldberg *et al.* 2022 [55] | AN, BN | 15 | 86 | Isreal | 275 | II, V |
| Graell *et al.* 2020 [56] | AN | 14 | 100 | Spain | 1818 | IV, V |
| Hansen *et al.* 2021 [57] | MIX | 15 and 28 | 98 | New Zealand | 121 | V |
| Haripersad *et al.* 2021 [58] | AN | N/K | N/K | Australia | 134 | I |
| Jones *et al.* 2020 [18] | MIX | N/K | N/K | Australia | 243 | V |
| Kersten *et al.* 2023 [59] | MIX | 0 to 18 | N/K | The Netherlands | 47621 | IV |
| Kim *et al.* 2021 [60] | MIX | 19 | 29 | USA | 8613 | I |
| Klump *et al.* 2022 [61] | BE | 22 | 100 | USA | 402 | VI |
| Machado *et al.* 2020 [62] | MIX | 28 | 95 | Portugal | 43 | I |
| Marino *et al.* 2021 [63] | AN | N/K | N/K | Italy | 1050 | I |
| Martinez de Quel *et al.* 2021 [64] | MIX | 35 | 37 | Spain | 161 | I, II |
| Matthews *et al.* 2021 [65] | AN | 15 | 83 | USA | 163 | V |
| Miskovic‐Wheatley *et al.* 2022 [15] | AN, BN | 25 | 92 | Australia | 1723 | II, VI |
| Özcan and Yeşilkaya 2021 [66] | AN, BN, EE | 32 | 73 | Turkey | 578 | VI |
| Richardson *et al.* 2020 [67] | MIX | N/K | 79 | Canada | 609 | II, IV, VI |
| Schelhorn *et al.* 2021 [68] | MIX | 34 | 71 | Germany | 3387 | I |
| Schlegl *et al.* 2020 (a) [69] | AN, BED | 24 | 100 | Germany | 159 | III |
| Schlegl *et al.* 2020 (b) [70] | BN, BED | 22 | 100 | Germany | 55 | III |
| Spettigue *et al.* 2021 [17] | AN, BN, BED | 15 | 74 | Canada | 91 | II, IV, V, VI |
| Spina *et al.* 2022 [71] | AN, BN, BED | 14 | 86 | Italy | 211 | V |
| Springall *et al.* 2021 [72] | AN, BN | 15 | 90 | Australia | 457 | II, IV, V |
| Surén *et al.* 2022 [73] | AN, BN, BED | 11 | 100 | Norway | 702035 | V |
| Takakura *et al.* 2022 [74] | MIX | 20 | 100 | Japan | 148 | II, V |
| Taquet *et al.* 2022 [75] | AN | 15 | 44 | USA | 18313 | I, IV |
| Tazeoglu et al., 2021 [76] | EE | 21 | 52 | Turkey | 386 | VI |
| Termorshuizen *et al.* 2020 [77] | AN, BN, BED | 31 | 98 | MIX | 1021 | III |
| Toulany *et al.* 2022 [78] | MIX | 10 | 49 | Canada | 3862051 | V |
| Trott *et al.* 2021 [79] | MIX | 36 | 84 | UK | 319 | I, II |
| Vuilier *et al.* 2021 [80] | AN, BN, BED | 30 | 64 | UK | 207 | III |

^1^ we report average age for included samples. In case these were not available, we report the reported range.

Abbreviations: AN, Anorexia Nervosa; BE, Binge Eating; BED, Binge Eating Disorders; BN, Bulimia Nervosa; EE, Emotional Eating; MIX, Mixed category of disorders; N/K, Not Known.

(I) changes in prevalence rates of AN, BN, and BED as assessed, (II) questionnaire data on changes in the severity of ED symptoms, (III) cross sectional data on self-reported changes in ED symptomatology (*e.g.,* pre-clinical symptoms), (IV) changes in comorbid mental health depression, anxiety and suicidality, (V) changes in need for care and access to care, age at admission, and length of stay, and (VI) questionnaire data on changes in EE and BE.

*Meta-analyses on patient data*

Random effects meta-analyses found an overall increase in prevalence rates of the ED’s during the pandemic, relative to those observed prior to the pandemic (**Table 2**). Bayesian meta-analysis confirmed this effect and showed very strong evidence favoring the alternative hypothesis over the null hypothesis of no effect (see **Table S4**, for Bayes Factors for the H_0_ and H_1_ and posterior probabilities). Given that virtually all samples were composed out of multiple types of EDs, we could not test whether the observed effect was due to a particular ED. Self-reported symptom prevalence rates were self-reported to be higher during the pandemic (see **Table 2** for prevalence rates self-reported in- and decreases in AN and BN symptoms). In fact, studies that directly assessed within-subject in- and decreases showed that the odds of self-reporting an increase in symptom prevalence relative to a decrease are high for AN (OR = 9.58, 95% CI = 1.09 to 80.45, *k* = 5, *N* = 1394) and BN (OR = 9.21, 95% CI = 1.41 to 60.64, *k* = 6, *N* = 1364). These findings also were confirmed in Bayesian met-analyses with Bayes Factors favoring H_1_ of 2504 and 2263 respectively.

AN, BN, and BED symptom severity were reported to be higher during the pandemic, but general severity was not (**Table 2**). Bayesian analyses provided anecdotal evidence favoring the null hypothesis of associations between the pandemic on ED symptom severity (see **Table S4**). Depression comorbidity increased significantly during the pandemic. Such effects were not observed for anxiety comorbidity and suicidality (see **Table 2**). In fact, for the latter outcome, results from Bayesian meta-analysis provided evidence for the H_0_ (**Table S4**).

Classical and Bayesian meta-analyses converged in their results on pandemic related changes in need for care and provide strong evidence that this increased during the pandemic. There were no significant associations between the pandemic and the average age at which patients sought care and duration of care. Bayesian meta-analyses on these two associations proved that the data is inconclusive, so without evidence for both the null and the alternative hypotheses (**Table 2** and **S4**).

*Meta-analyses in non-patient samples*

Our data show that both BE and EE increased in the general population samples, but only the change in BE reached statistical significance (**Table 2**). Bayesian meta-analyses show that the evidential strength for the existence of these associations is anecdotal to moderate (**Table S4**).

**Table 2.** Mental health disorder and COVID-19 course variables from frequentist meta-analysis.

| ***ED patient data*** |  |  |  |  |  |
| --- | --- | --- | --- | --- | --- |
| ***Prevalence rates ED*** | **OR (95% CI)** | ***k*** | ***N*** | ***I^2^*** | **Egger’s test** |
| **AN / MIX ^1^** | **1.30 (1.05 to 1.54) ***** | **10** | **33032** | **80.7 ***** | **0.77** |
| ***ED symptom prevalence*** | **Proportion (95% CI)** | ***k*** | ***N*** | ***I^2^*** | **Egger’s test** |
| **AN increase symptom prevalence** | **0.59 (0.39 to 0.80)** | **7** | **1483** | **98.7 ***** | **-0.87** |
| **BN increase symptom prevalence** | **0.58 (0.42 to 0.74)** | **7** | **1376** | **91.9 ***** | **-1.30** |
| **AN decrease symptom prevalence** | **0.23 (0.09 to 0.37)** | **5** | **1394** | **98.1 ***** | **0.66** |
| **BN decrease symptom prevalence** | **0.24 (0.13 to 0.38)** | **6** | **1346** | **97.0 ***** | **0.34** |
| ***ED symptom severity*** | **Cohen’s *d* (95% CI)** | ***k*** | ***N*** | ***I^2^*** | **Egger’s test** |
| **ED severity score** | **0.11 (-0.06 to 0.28)** | **11** | **1875** | **86.8 ***** | **0.56** |
| **AN severity** | **0.29 (0.12 to 0.46) **** | **7** | **3095** | **50.7 ***** | **1.43** |
| **BN severity** | **0.24 (0.03 to 0.45) ***** | **7** | **2873** | **80.5 ***** | **1.73** |
| **BED severity** | **0.27 (0.02 to 0.43) *** | **4** | **2243** | **49.4** | **1.38** |
| ***Comorbid psychiatric symptoms*** | **Cohen’s *d* (95% CI)** | ***k*** | ***N*** | ***I^2^*** | **Egger’s test** |
| ***Depression*** | **0.21 (0.07 to 0.35) **** | **10** | **2012** | **67.6 **** | **0.23** |
| ***Anxiety*** | **0.23 (-0.01 to 0.47)** | **9** | **1970** | **0.10** | **0.17** |
| ***Suicidality*** | **0.10 (-0.42 to 0.61)** | **3** | **8722** | **41.4** | **0.16** |
| ***Need for care and impairment*** | **OR (95% CI)** | ***k*** | ***N*** | ***I^2^*** | **Egger’s test** |
| **Mix care category ^2^** | **1.74 (1.48 to 2.00) ***** | **13** | **5260823** | **83.3***** | **3.64 ** ^3^** |
| **Hospitalization** | **1.98 (1.37 to 1.48) ***** | **4** | **4862668** | **56.8** | **1.24** |
|  | **Cohen’s *d* (95% CI)** | ***k*** | ***N*** | ***I^2^*** | **Egger’s test** |
| **Length of stay** | **0.19 (-0.47 to 0.85)** | **5** | **1099** | **95.4***** | **-0.42** |
| **Age at admission** | **-0.31 (-0.04 to 0.61)** | **5** | **837** | **0.00** | **0.56** |
| ***General population data*** |  |  |  |  |  |
| ***ED related behaviors ^1^*** | **Cohen’s *d* (95% CI)** | ***k*** | ***N*** | ***I^2^*** | **Egger’s test** |
| **Binge eating** | **0.14 (0.02 to 0.22) *** ^2^** | **9** | **4882** | **0.0** | **2.48 * ^4^** |
| **Emotional eating** | **1.05 (-0.03 to 2.75)** | **6** | **1091** | **99.0***** | **-0.61** |

* *P* < 0.05, ** *P* < 0.01, *** *P* < 0.001

^1^ All studies, except for 2, reported prevalence rates for mixed ED categories. Hence no analyses by ED subtypes were performed.

^2^ Most often a mix of emergency visits, planned need for expert care, and hospitalization.

^3^ Trim and fill analysis yielded a somewhat smaller, yet significant, effect size estimate (OR = 1.65, 95% CI = 1.40 to 1.89).

^4^ Trim and fill analysis yielded a somewhat smaller, yet significant, effect size estimate (*d* = 0.12, 95% CI = 0.60 to 0.18).

*Abbreviations*. AN, Anorexia Nervosa: BED, Binge Eating Disorder; BN, Bulimia Nervosa; ED, Eating Disorder; *I^2^*, amount of between-study heterogeneity; *k*, number of effect-size estimates; *N*, number of participants; OR, Odds Ratio.

*Between-study heterogeneity, publication bias and effect-moderators,*

Between-study heterogeneity was observed in basically each meta-analysis that we performed. There were no moderators that consistently explained variance in this heterogeneity (**Table S5**). We were not able to assess effects of timing in the pandemic, since there was hardly any variation in this variable (almost all data was gathered early in the pandemic). There was no consistent evidence for the existence of publication bias. Correcting effect-size estimates and 95% CI’s for the potential effects of publication bias largely yielded similar results and in no case warranted categorical different conclusions the data (**Table 2**).

**Discussion**

The present meta‐analysis pooled 46 independent studies on a total of 4.688.559 subjects. Age range of the samples in the included studies was 7 to 65 and in a majority of cases, female participants were overrepresented. Data predominantly were gathered in the USA, southern and northern Europe and Australia (41 of 46 samples). Our results show that the prevalence of the ED and their symptoms increased during the COVID-19 pandemic relative to time frames prior to this. Symptom severity of AN and comorbid depression, but not anxiety and suicidality, were also found to be higher during the pandemic. Concordantly, we found that hospitalization, emergency department visits and in-patient admission rates were found to be higher during the pandemic. We report, with moderate strength of evidence, that in the general population, BE behavior was reported more often in times of the pandemic. We did not observe such increases in EE behavior. With regard to this latter null finding, it should be noted that the results were influenced by an outlier in the data. Freitas *et al.* [53] reported a decrease in EE during the pandemic, relative to prior to the pandemic. This study, however, applied different measurements pre- and during the pandemic (interview versus self-report respectively), which could have driven the unsuspected results. When we excluded this study from our meta-analysis, EE symptoms were observed to be higher during the Covid-19 pandemic (*p* < .01, BF favoring H_1_ = 3.48). There were no consistent associations between the prespecified moderators and there was little evidence for publication bias. We were not able to investigate timing of the pandemic on outcome because there was too little variance in this variable to allow analysis.

This was not a study that investigated mechanisms or mediators, yet we do have some hypotheses that could explain the pattern of results that we obtained. First of all, the pandemic and resulting quarantines, restrictions and social isolation may have increased feelings of stress, ineffectiveness, loss of control, fear and boredom [81-83]. EDs have often been conceptualized as maladaptive responses to stressful situations [84, 85], that could temporarily decrease awareness and intensity of the aversive emotions [54, 86, 87]. Second, the pandemic induced disruptions in daily routines caused changes in usual patterns of physical activity, eating, and sleeping routines [25, 88-90]. This may have brought forth concerns of gaining weight, which is common in people with EDs [25, 91], and increased the symptoms of EDs [90] and related behaviors such as EE. Third, the immense increase in social media usage during the pandemic may have led to raised worries regarding body appearance and disordered eating. Fourth, limiting grocery trips and food access may have resulted in food insecurity, worries about the availability of food, and hoarding that are related to urges to binge [12, 27]. A fifth potential explanation of our findings is in term of limited access to professional services and social sources, due to lockdowns [77, 90].

Our findings are largely similar to those reported by previous systematic reviews and meta-analyses [12, 19-27]. Yet our work stands out from them for several reasons. First of all, we report results on a multitude of data for most associations. We also are able to pool data on more specific outcome types (*e.g.,* ED related hospitalization and EE). Furthermore, our work stands out by applying both classical null hypothesis significance testing and Bayesian approaches to meta-analyses, and this has proved advantages (*e.g.,* in this we can present evidence for the null hypothesis that the severity of general ED symptoms did not increase in patients during the pandemic). Another strength of our work is that article selection and data extraction were performed by three researchers independently. Another strength is that we pooled data across studies on a small variety of standardized assessment instruments. Limitations of our study include, but are not limited to, the observational nature of our input data. Included studies mainly used convenience sampling that for the larger part relied on self-report of the participants with no follow-up data. Future studies could account for the bias that this might have caused. Future meta-analyses also could compare the results of different methods by which samples were recruited for studies on the impact of the pandemic on ED’s. There were certain limitations with the generalizability of the included studies. For instance, the mean percentage of females was high. Another limitation of our work is that we only could marginally look into the effects of timing in the pandemic and its associations with ED related psychopathology. From all included studies we extracted an effect size reflecting a change in ED related psychopathology pre versus during the pandemic. This is informative, but does not capture the course and the dynamics of symptom development and alleviation. The data reported Hill *et al.* [95] show this perfectly with large monthly variation in suicidal ideation and behavior within the pandemic.

Future studies should elaborately investigate the moderating factors for the development and worsening of AN and BN symptomology in specific timeframes. Publication bias seemed evident in the analysis on the broad outcome category ‘need for care’. Adjusting for this by means of trim and fill methods led to somewhat lower effect size estimates and a wider confidence interval, yet they remained statistically significant. The Bayes Factor also strongly supported the alternative hypothesis of an effect of the pandemic on need for care over the null hypothesis. Finally, our search terms may have been limited because of the restricted number of search terms. However, some rather broad terms were used (*e.g.,* eating) and we only included about 1.5% of the initial hits. A reviewer suggested using some more specific terms *a.o.,* compulsive exercise. This, we found in a pilot, resulted in significant more initial hits, but not to more eligible articles. Probably, a term as *compulsive exercise* is too generic, leading to many hits in for the current work irrelevant domains (*e.g.,* obsessive compulsive disorder or physical activity).

The findings reported in this current systematic review have some clinical implications. The prevalence and/or severity of ED symptoms were found to be increased during the Covid-19 period, suggesting the need to develop intervention and prevention programs for EDs. Such methods preferably are available for a broad range of ED related symptoms and disorders, and accessible via flexible and easy to rollout web-conferencing software. Even more accessible and proven to be useful, also in times of the pandemic, are freely available apps promoting self-monitoring and teaching coping skills [96]. Furthermore, public health policies and intervention programs should be suitable in case of confinement or related circumstances. Future single studies and meta-analyses could investigate this, together with a focus on moderating effects of age, gender and culture.

To the best of our knowledge, this is the first systematic review with classical and Bayesian meta-analysis that assessed the effect of the Covid-19 pandemic on ED psychopathology and severity, ED related need for care, psychiatric comorbidity, and BE and EE. We show that the COVID-19 pandemic is associated with a wide spread and worrisome negative effect on ED pathology and ED-related need for care.

*What is already known on this subject?*

The COVID-19 pandemic is suspected to have had a negative effect on the prevalence rates of, and indicators for, ED psychopathology. What is unknown is the range and the magnitude of this effect.

*What does this study add?*

Our data suggest that in the face of the COVID-19 pandemic, ED prevalence rates, their symptoms, psychiatric comorbidity, and need for care increased. The evidential strength on the existence of these associations ranges from anecdotal (*e.g.,* the pandemic related increase in emotional eating in general population samples) to very strong (*e.g.,* the pandemic related increase in ED related need for care).

*Data availability*

*The data that we gathered and generated in this project is available for other projects upon reasonable request.*

**References**

1. Giannis D, Ziogas IA, Gianni P. Coagulation disorders in coronavirus infected patients: COVID-19, SARS-CoV-1, MERS-CoV and lessons from the past. J Clin Virol. 2020 Jun 1;127:104362.
2. Wong G, Bi YH, Wang QH, Chen XW, Zhang ZG, Yao YG. Zoonotic origins of human coronavirus 2019 (HCoV-19/SARS-CoV-2): why is this work important? Zoological Res. 2020 May 18;41(3):213.
3. *WHO Coronavirus (COVID-19) Dashboard*. (2022). With Vaccination Data. Retrieved July 1, 2022, from <https://covid19.who.int/>
4. Robinson E, Sutin AR, Daly M, Jones A. A systematic review and meta-analysis of longitudinal cohort studies comparing mental health before versus during the COVID-19 pandemic in 2020. J Affect Disord. 2022 Jan 1;296:567-76.
5. Samji H, Wu J, Ladak A, Vossen C, Stewart E, Dove N, Long D, Snell G. Mental health impacts of the COVID‐19 pandemic on children and youth–a systematic review. Child Adoles Mental Health. 2022 May;27(2):173-89.
6. Todisco P, Donini LM. Eating disorders and obesity (ED&O) in the COVID-19 storm. Eat Weight Disord. 2021 *26*(3), 747-750.
7. Troop NA, Holbrey A, Treasure JL. Stress, coping, and crisis support in eating disorders. International J Eating Disord. 1998 Sep;24(2):157-66.
8. Vartanian LR, Smyth JM, Zawadzki MJ, Heron KE, Coleman SR. Early adversity, personal resources, body dissatisfaction, and disordered eating. Int J Eating Disordd. 2014 Sep;47(6):620-9.
9. Molendijk ML, Hoek HW, Brewerton TD, Elzinga BM. Childhood maltreatment and eating disorder pathology: a systematic review and dose-response meta-analysis. Psychol Med. 2017 Jun;47(8):1402-16.
10. Burton AL, Abbott MJ. Conceptualising binge eating: a review of the theoretical and empirical literature. Beh Change. 2017 Sep;34(3):168-98.
11. Wallis DJ, Hetherington MM. Stress and eating: the effects of ego-threat and cognitive demand on food intake in restrained and emotional eaters. Appetite. 2004 Aug 1;43(1):39-46.
12. Miniati M, Marzetti F, Palagini L, Marazziti D, Orrù G, Conversano C, Gemignani A. Eating disorders spectrum during the COVID pandemic: A systematic review. Front Psychol. 2021:4161.
13. Modrzejewska A, Czepczor-Bernat K, Modrzejewska J, Matusik P. Eating motives and other factors predicting emotional overeating during COVID-19 in a sample of Polish adults. Nutrients. 2021 May 13;13(5):1658.
14. Baenas I, Etxandi M, Munguía L, Granero R, Mestre-Bach G, Sánchez I, Ortega E, Andreu A, Moize VL, Fernández-Real JM, Tinahones FJ. Impact of COVID-19 lockdown in eating disorders: a multicentre collaborative international study. Nutrients. 2021 Dec 27;14(1):100.
15. Miskovic-Wheatley J, Koreshe E, Kim M, Simeone R, Maguire S. The impact of the COVID-19 pandemic and associated public health response on people with eating disorder symptomatology: an Australian study. J Eating Disord. 2022 Dec;10(1):1-4.
16. Albert U, Losurdo P, Leschiutta A, Macchi S, Samardzic N, Casaganda B, de Manzini N, Palmisano S. Effect of SARS-CoV-2 (COVID-19) pandemic and lockdown on body weight, maladaptive eating habits, anxiety, and depression in a bariatric surgery waiting list cohort. Obesity Surgery. 2021 May;31(5):1905-11.
17. Spettigue W, Obeid N, Erbach M, Feder S, Finner N, Harrison ME, Isserlin L, Robinson A, Norris ML. The impact of COVID-19 on adolescents with eating disorders: a cohort study. J Eat Disord. 2021 Dec;9(1):1-8.
18. Jones PD, Gentin A, Clarke J, Arakkakunnel J. Fewer respiratory admissions in COVID‐19 era. J Paediatrics Child Health. 2020 Dec;56(12):1997-9.
19. Linardon J, Messer M, Rodgers RF, Fuller‐Tyszkiewicz M. A systematic scoping review of research on COVID‐19 impacts on eating disorders: A critical appraisal of the evidence and recommendations for the field. Int J Eat Disord. 2022 Jan;55(1):3-8.
20. Monteleone AM, Cascino G, Barone E, Carfagno M, Monteleone P. COVID-19 pandemic and eating disorders: What can we learn about psychopathology and treatment? A systematic review. Current Psychiatry Res. 2021 Dec;23(12):1-5
21. Neira C, Godinho R, Rincón F, Mardones R, Pedroso J. Consequences of the covid-19 syndemic for nutritional health: A systematic review. Nutrients. 2021 Apr 1;13(4):1168.
22. Devoe J, Han D, Anderson A, Katzman A, Patten DK, Soumbasis SB, Flanagan J, Paslakis G, Vyver E, Marcoux G, Dimitropoulos G. The impact of the COVID‐19 pandemic on eating disorders: A systematic review. Int J Eat Disord. 2022 Apr.
23. McLean, C. P., Utpala, R., & Sharp, G. (2022). The impacts of COVID-19 on eating disorders and disordered eating: A mixed studies systematic review and implications. *Front Psychol*, *13*.
24. Sideli L, Lo Coco G, Bonfanti RC, Borsarini B, Fortunato L, Sechi C, Micali N. Effects of COVID‐19 lockdown on eating disorders and obesity: A systematic review and meta‐analysis. European Eat Disord Rev. 2021 Nov;29(6):826-41.
25. Haghshomar M, Shobeiri P, Brand S, Rossell SL, Akhavan Malayeri A, Rezaei N. Changes of symptoms of eating disorders (ED) and their related psychological health issues during the COVID-19 pandemic: a systematic review and meta-analysis. J Eat Disord. 2022 Dec;10(1):1-8.
26. Khraisat BR, Al-Jeady AM, Alqatawneh DA, Toubasi AA, AlRyalat SA. The prevalence of mental health outcomes among eating disorder patients during the COVID-19 pandemic: A meta-analysis. Clin Nutrition ESPEN. 2022 Apr 1;48:141-7.
27. Gao Y, Bagheri N, Furuya-Kanamori L. Has the COVID-19 pandemic lockdown worsened eating disorders symptoms among patients with eating disorders? A systematic review. J Public Health. 2022 1-10.
28. Herle M, De Stavola B, Hübel C, Abdulkadir M, Ferreira DS, Loos RJ, Micali N. A longitudinal study of eating behaviours in childhood and later eating disorder behaviours and diagnoses. The Br J Psychiatry. 2020 216(2), 113-119.
29. Keysers C, Gazzola V, Wagenmakers EJ. Using Bayes factor hypothesis testing in neuroscience to establish evidence of absence. Nature Neurosci. 2020 Jun 23, 788–799.
30. Stroup DF, Berlin JA, Morton SC, Olkin I, Williamson GD, Rennie D, Moher D, Becker BJ, Sipe TA, Thacker SB. Meta-analysis of observational studies in epidemiology: a proposal for reporting. JAMA. 2000 Apr 19;283(15):2008-12.
31. Moher D, Liberati A, Tetzlaff J, Altman DG, PRISMA Group (2009) Preferred reporting items for systematic reviews and meta-analyses: the PRISMA statement. PLoS Med. <https://doi.org/10.1371/journal.pmed.1000097>
32. Lievense AM, Bierma‐Zeinstra SM, Verhagen AP, Van Baar ME, Verhaar JA, Koes BW. Influence of obesity on the development of osteoarthritis of the hip: a systematic review. Rheumatol. 2002 Oct 1;41(10):1155-62.
33. StataCorp. Stata Statistical Software: Release 17. College Station, TX: StataCorp LLC. 2017.
34. Metelli S, Chaimani A. Challenges in meta-analyses with observational studies. Evidence-based Mental Health. 2020 May 1;23(2):83-7.
35. Sterne JA, Egger M, Smith GD. Investigating and dealing with publication and other biases in meta-analysis. BMJ. 2001 Jul 14;323(7304):101-5.
36. Morey RD, Rouder JN. Bayes factor approaches for testing interval null hypotheses. Psychol Methods. 2011 Dec;16(4):406.
37. Volpe U, Tortorella A, Manchia M, Monteleone AM, Albert U, Monteleone P. Eating disorders: what age at onset? Psychiatry Res. 2016 238, 225-227.
38. Simone M, Telke S, Anderson LM, Eisenberg M, Neumark-Sztainer D. Ethnic/racial and gender differences in disordered eating behavior prevalence trajectories among women and men from adolescence into adulthood. Soc Sci Med. 2022 294, 114720.
39. Jung RG, Di Santo P, Clifford C, Prosperi-Porta G, Skanes S, Hung A, Hibbert B. Methodological quality of COVID-19 clinical research. Nature Communications. 2021 12(1), 1-10.
40. Allgaier K, Schneider PS, Buck S, Reusch PA, Hagmann D, Barth GM, Renner TJ. Child and Adolescent Psychiatric Emergencies During the Second Wave of the SARS-CoV2-19 Pandemic: Findings from the Tübingen University Hospital. Zeitschrift fur Kinder-und Jugendpsychiatrie und Psychotherapie. 2022 Feb 28.
41. Branley-Bell D, Talbot CV. Exploring the impact of the COVID-19 pandemic and UK lockdown on individuals with experience of eating disorders. J Eat Disord. 2020 Dec;8(1):1-2.
42. Bianchi D, Baiocco R, Pompili S, Lonigro A, Di Norcia A, Cannoni E, Longobardi E, Zammuto M, Di Tata D, Laghi F. Binge Eating and Binge Drinking in Emerging Adults During COVID-19 Lockdown in Italy: An Examination of Protective and Risk Factors. Emerging Adulthood. 2022 Feb;10(1):291-303.
43. Breiner CE, Miller ML, Hormes JM. Changes in eating and exercise behaviors during the COVID-19 pandemic in a community sample: A retrospective report. Eat Behav. 2021 Aug 1;42:101539.
44. Carison A, Babl FE, O'Donnell SM. Increased paediatric emergency mental health and suicidality presentations during COVID‐19 stay at home restrictions. Emergency Med Australasia. 2022 Feb;34(1):85-91.
45. Castellini G, Cassioli E, Rossi E, Innocenti M, Gironi V, Sanfilippo G, Felciai F, Monteleone AM, Ricca V. The impact of COVID‐19 epidemic on eating disorders: A longitudinal observation of pre versus post psychopathological features in a sample of patients with eating disorders and a group of healthy controls. Int J Eat Disord. 2020 Nov;53(11):1855-62.
46. Christensen KA, Forbush KT, Richson BN, Thomeczek ML, Perko VL, Bjorlie K, Christian K, Ayres J, Wildes JE, Mildrum Chana S. Food insecurity associated with elevated eating disorder symptoms, impairment, and eating disorder diagnoses in an American University student sample before and during the beginning of the COVID‐19 pandemic. Int J Eat Disord. 2021 Jul;54(7):1213-23.
47. Colleluori G, Goria I, Zillanti C, Marucci S, Dalla Ragione L. Eating disorders during COVID-19 pandemic: the experience of Italian healthcare providers. Eat Weight Disord. 2021 Dec;26(8):2787-93.
48. Datta N, Van Wye E, Citron K, Matheson B, Lock JD. The COVID-19 pandemic andyouth with anorexia nervosa: A retrospective comparative cohort design. Int J Eat Disord. 2022 Sep 20:10.1002/eat.23817. doi: 10.1002/eat.23817.
49. Elmacıoğlu F, Emiroğlu E, Ülker MT, Kırcali BÖ, Oruç S. Evaluation of nutritional behaviour related to COVID-19. Public Health Nutrition. 2021 Feb;24(3):512-8.
50. Eray S, Sahin V. Covid-19 Pandemic may have Unique Effects on Emergency Admissions for Pediatric Psychopathology: A Single-Center Study. Psychiatry Behav Sci. 2021 Jun 20;11(2):115.
51. Favreau M, Hillert A, Osen B, Gärtner T, Hunatschek S, Riese M, Hewera K, Voderholzer U. Psychological consequences and differential impact of the COVID-19 pandemic in patients with mental disorders. Psychiatry Res. 2021 Aug; 302:114045. doi: 10.1016/j.psychres.2021.114045.
52. Feldman MA, King CK, Vitale S, Denhardt B, Stroup S, Reese J, Stromberg S. The impact of COVID-19 on adolescents with eating disorders: Increased need for medical stabilization and decreased access to care. Int J Eat Disord. 2022 Jul 30:10.1002/eat.23788. doi: 10.1002/eat.23788.
53. Freitas FD, de Medeiros AC, Lopes FD. Effects of social distancing during the COVID-19 pandemic on anxiety and eating behavior—a longitudinal study. Front Psychol. 2021 Jun 1;12:645754.
54. Giel KE, Schurr M, Zipfel S, Junne F, Schag K. Eating behaviour and symptom trajectories in patients with a history of binge eating disorder during COVID‐19 pandemic. Eur Eat Disord Rev. 2021 Jul;29(4):657-62.
55. Goldberg L, Ziv A, Vardi Y, Hadas S, Zuabi T, Yeshareem L, Gur T, Steinling S, Scheuerman O, Levinsky Y. The effect of COVID-19 pandemic on hospitalizations and disease characteristics of adolescents with anorexia nervosa. Eur J Pediatrics. 2022 Apr;181(4):1767-71.
56. Graell M, Morón‐Nozaleda MG, Camarneiro R, Villaseñor Á, Yáñez S, Muñoz R, Martínez‐Núñez B, Miguélez‐Fernández C, Muñoz M, Faya M. Children and adolescents with eating disorders during COVID‐19 confinement: Difficulties and future challenges. Eur Eat Disord Rev. 2020 Nov;28(6):864-70.
57. Hansen SJ, Stephan A, Menkes DB. The impact of COVID-19 on eating disorder referrals and admissions in Waikato, New Zealand. J Eat Disord. 2021 Dec;9(1):1-0.
58. Haripersad, Y. V., Kannegiesser-Bailey, M., Morton, K., Skeldon, S., Shipton, N., Edwards, K., Newton, R., Newell, A., Stevenson, P. G., & Martin, A. C. (2021). Outbreak of anorexia nervosa admissions during the COVID-19 pandemic. *Archives Dis Childhood*, *106*(3), e15. <https://doi.org/10.1136/archdischild-2020-319868>
59. Kersten JM, van Veen M, van Houten MA, Wieringa J, Noordzij JG, Bekhof J, Tramper-Stranders GA, Visser-Trip H, VetNJ, Kruizinga MD. Adverse effect of lockdowns during the COVID-19 pandemic: increased incidence of pediatric crisis admissions due to eating disorders and adolescent intoxications. Eur J Pediatrics, 2023 1-6.
60. Kim HH, Jung JH. Social isolation and psychological distress during the COVID-19 pandemic: A cross-national analysis. Gerontologist. 2021 Feb;61(1):103-13.
61. Klump, K. L., Mikhail, M. E., Anaya, C., Fowler, N., Neale, M., Keel, P. K., ... & Burt, S. A. (2022). The effects of the COVID-19 pandemic on disordered eating symptoms in women: A 49-day, daily study before and during the outbreak in the United States. *J Psychopathol Clin Sci*.
62. Machado PP, Pinto-Bastos A, Ramos R, Rodrigues TF, Louro E, Gonçalves S, Brandão I, Vaz A. Impact of COVID-19 lockdown measures on a cohort of eating disorders patients. J Eat Disord. 2020 Dec;8(1):1-8.
63. Marino A, Gliubizzi C, Reina F, Nocera GM, Marchese F, Trapolino E. Increase in admissions for anorexia nervosa after lockdown measures: Focus on a children's neuropsychiatry unit. Gen Hosp Psychiatry. 2021 Jun 17.
64. Martínez-de-Quel Ó, Suárez-Iglesias D, López-Flores M, Pérez CA. Physical activity, dietary habits and sleep quality before and during COVID-19 lockdown: A longitudinal study. Appetite. 2021 Mar 1;158:105019.
65. Matthews A, Kramer RA, Peterson CM, Mitan L. Higher admission and rapid readmission rates among medically hospitalized youth with anorexia nervosa/atypical anorexia nervosa during COVID-19. Eat Beh. 2021 Dec 1;43:101573.
66. Özcan BA, Yeşİlkaya B. Adverse Effect of Emotional Eating Developed During the COVID-19 Pandemic on Healthy Nutrition, a Vicious Circle: A cross-sectional descriptive study. Revista Española de Nutrición Humana y Dietética. 2021 Oct 10;25:e1144-.
67. Richardson C, Patton M, Phillips S, Paslakis G. The impact of the COVID-19 pandemic on help-seeking behaviors in individuals suffering from eating disorders and their caregivers. Gen Hosp Psychiatry. 2020 Nov 1;67:136-40.
68. Schelhorn I, Ecker A, Lüdtke MN, Rehm S, Tran T, Bereznai JL, Meyer ML, Sütterlin S, Kinateder M, Lugo RG, Shiban Y. Psychological Burden During the COVID-19 Pandemic in Germany. Front Psychol. 2021;12.
69. Schlegl S, Meule A, Favreau M, Voderholzer U. Bulimia nervosa in times of the COVID‐19 pandemic—Results from an online survey of former inpatients. Eur Eat Disord Rev. 2020 Nov;28(6):847-54.
70. Schlegl S, Maier J, Meule A, Voderholzer U. Eating disorders in times of the COVID‐19 pandemic—Results from an online survey of patients with anorexia nervosa. Int J Eat Disord. 2020 Nov;53(11):1791-800.
71. Spina G, Roversi M, Marchili MR, Raucci U, Fini F, Mirra G, Testa G, Guarnieri B, Clemente A, Diamanti A, Zanna V, Castiglioni MA, Vicari S, Reale A, Villani A. Psychiatric comorbidities and dehydration are more common in children admitted to the emergency department for eating disorder in the COVID-19 era. Eat Weight Disord. 2022. 1-8. AOP.
72. Springall G, Cheung M, Sawyer SM, Yeo M. Impact of the coronavirus pandemic on anorexia nervosa and atypical anorexia nervosa presentations to an Australian tertiary paediatric hospital. J Pediatrics Child Health. 2022 Mar;58(3):491-6.
73. Surén P, Skirbekk AB, Torgersen L, Bang L, Godøy A, Hart RK. Eating Disorder Diagnoses in Children and Adolescents in Norway Before vs During the COVID-19 Pandemic. Eating disorder diagnoses in children and adolescents in Norway before versus during the pandemic. JAMA network open. 2022.
74. Takakura S, Toda K, Yamashita M, Kitajima T, Suematsu T, Yokoyama H, Sudo, N. Potential impact of the COVID-19 pandemic on japanese patients with eating disorders-a cross-sectional study. *BioPsychoSocial Med*. 2022 *16*(1), 1-6.
75. Taquet M, Geddes JR, Luciano S, Harrison PJ. Incidence and outcomes of eating disorders during the COVID-19 pandemic. Br J Psychiatry. 2022 May;220(5):262-4.
76. Tazeoglu A, Bozdogan FB, Idiz C. Evaluation of osmaniye korkut ata university students’ eating behaviors during the quarantine period during the covid-19 pandemic period. Nutrición Clínica y Dietética Hospitalaria. 2021;41(2):86-93.
77. Termorshuizen JD, Watson HJ, Thornton LM, Borg S, Flatt RE, MacDermod CM, Harper LE, van Furth EF, Peat CM, Bulik CM. Early impact of COVID‐19 on individuals with self‐reported eating disorders: A survey of~ 1,000 individuals in the United States and the Netherlands. International Journal of Eating Disorders. 2020 Nov;53(11):1780-90.
78. Toulany A, Kurdyak P, Guttmann A, Stukel TA, Fu L, Strauss R, Fiksenbaum L, Saunders NR. Acute care visits for eating disorders among children and adolescents after the onset of the COVID-19 pandemic. Journal of Adolescent Health. 2022 Jan 1;70(1):42-7.
79. Trott M, Johnstone J, Pardhan S, Barnett Y, Smith L. Changes in body dysmorphic disorder, eating disorder, and exercise addiction symptomology during the COVID-19 pandemic: A longitudinal study of 319 health club users. Psychiatry Res. 2021 Apr 1;298:113831.
80. Vuillier L, May L, Greville-Harris M, Surman R, Moseley RL. The impact of theCOVID-19 pandemic on individuals with eating disorders: the role of emotion regulation and exploration of online treatment experiences. J Eat Disord. 2021 Jan 12;9(1):10. doi: 10.1186/s40337-020-00362-9.
81. Chao M, Chen X, Liu T, Yang H, Hall BJ. Psychological distress and state boredom during the COVID-19 outbreak in China: the role of meaning in life and media use. Eur J Psychotraumatol. 2020 Dec 31;11(1):1769379.
82. Husky MM, Kovess-Masfety V, Swendsen JD. Stress and anxiety among university students in France during Covid-19 mandatory confinement. Comprehen Psychiatry. 2020 Oct 1;102:152191.
83. Shah SM, Mohammad D, Qureshi MF, Abbas MZ, Aleem S. Prevalence, psychological responses and associated correlates of depression, anxiety and stress in a global population, during the coronavirus disease (COVID-19) pandemic. Community Mental Health J. 2021 Jan;57(1):101-10.
84. Steinhausen HC, editor. Eating disorders in adolescence: Anorexia and bulimia nervosa. Walter de Gruyter GmbH & Co KG; 2020 Oct 12.
85. Eisler I, Dare C, Hodes M, Russell G, Dodge E, Le Grange D. Family therapy for adolescent anorexia nervosa: The results of a controlled comparison of two family interventions. J Child Psychol Psychiatry Allied Disciplines. 2000 Sep;41(6):727-36.
86. Burton AL, Hay P, Kleitman S, Smith E, Raman J, Swinbourne J, Touyz SW, Abbott MJ. Confirmatory factor analysis and examination of the psychometric properties of the eating beliefs questionnaire. BMC Psychiatry. 2017 Jul 3;17(1):237. doi: 10.1186/s12888-017-1394-z.
87. Wallis DJ, Hetherington MM. Emotions and eating. Self-reported and experimentally induced changes in food intake under stress. Appetite. 2009 Apr;52(2):355-62. doi: 10.1016/j.appet.2008.11.007.
88. McLean C, Utpala R, Sharp G. The impacts of COVID-19 on eating disorders and disordered eating: A mixed studies systematic review and implications for healthcare professionals, carers, and self. AOP.
89. Phillipou A, Meyer D, Neill E, Tan EJ, Toh WL, Van Rheenen TE, Rossell SL. Eating and exercise behaviors in eating disorders and the general population during the COVID‐19 pandemic in Australia: Initial results from the COLLATE project. Int J Eat Disord. 2020 Jul;53(7):1158-65.
90. Rodgers RF, Lombardo C, Cerolini S, Franko DL, Omori M, Fuller‐Tyszkiewicz M, Linardon J, Courtet P, Guillaume S. The impact of the COVID‐19 pandemic on eating disorder risk and symptoms. Int J Eat Disord. 2020 Jul;53(7):1166-70.
91. Walsh O, McNicholas F. Assessment and management of anorexia nervosa during COVID-19. Ir J Psychological Med. 2020 Sep;37(3):187-91.
92. Gobin KC, Mills JS, McComb SE. The Effects of the COVID-19 Pandemic Lockdown on Eating, Body Image, and Social Media Habits Among Women With and Without Symptoms of Orthorexia Nervosa. Front Psychol. 2021;12.
93. Price M, Legrand AC, Brier ZM, van Stolk-Cooke K, Peck K, Dodds PS, Danforth CM, Adams ZW. Doomscrolling during COVID-19: The negative association between daily social and traditional media consumption and mental health symptoms during the COVID-19 pandemic. Psychological Trauma: Theory Res Practice Policy. 2022 Feb 14.
94. Robertson M, Duffy F, Newman E, Bravo CP, Ates HH, Sharpe H. Exploring changes in body image, eating and exercise during the COVID-19 lockdown: A UK survey. Appetite. 2021 Apr 1;159:105062.
95. Hill RM, Rufino K, Kurian S, Saxena J, Saxena K, Williams L. Suicide Ideation and Attempts in a Pediatric Emergency Department Before and During COVID-19. Pediatrics. 2021 Mar;147(3):e2020029280. doi: 10.1542/peds.2020-029280.
96. Cooper M, Reilly EE, Siegel JA, Coniglio K, Sadeh-Sharvit S, Pisetsky EM, Anderson LM. Eating disorders during the COVID-19 pandemic and quarantine: an overview of risks and recommendations for treatment and early intervention. Eat Disord. 2022 *30*(1), 54-76.

Page 2 of 2
